# Lactic acid separation and recovery from fermentation broth by ion-exchange resin: A review

**DOI:** 10.1186/s40643-021-00384-4

**Published:** 2021-04-20

**Authors:** Nur Akmal Solehah Din, Seng Joe Lim, Mohamad Yusof Maskat, Sahilah Abd Mutalib, Nurul Aqilah Mohd Zaini

**Affiliations:** 1 Department of Food Sciences, Faculty of Science and Technology, Universiti Kebangsaan Malaysia, UKM, 43600 Bangi, Selangor Malaysia; 2 Innovation Centre for Confectionery Technology (MANIS), Faculty of Science and Technology, Universiti Kebangsaan Malaysia, UKM, 43600 Bangi, Selangor Malaysia

**Keywords:** Lactic acid, Fermentation, Ion-exchange resin, In-site separation, Recovery

## Abstract

Lactic acid has become one of the most important chemical substances used in various sectors. Its global market demand has significantly increased in recent years, with a CAGR of 18.7% from 2019 to 2025. Fermentation has been considered the preferred method for producing high-purity lactic acid in the industry over chemical synthesis. However, the recovery and separation of lactic acid from microbial fermentation media are relatively complicated and expensive, especially in the process relating to second-generation (2G) lactic acid recovery. This article reviews the development and progress related to lactic acid separation and recovery from fermentation broth. Various aspects are discussed thoroughly, such as the mechanism of lactic acid production through fermentation, the crucial factors that influence the fermentation process, and the separation and recovery process of conventional and advanced lactic acid separation methods. This review's highlight is the recovery of lactic acid by adsorption technique using ion-exchange resins with a brief focus on the potential of in-site separation strategies alongside the important factors that influenced the lactic acid recovery process by ion exchange. Apart from that, other lactic acid separation techniques, such as chemical neutralization, liquid–liquid extraction, membrane separation, and distillation, are also thoroughly reviewed.

## Introduction

The majority of industrial production of lactic acid today relies on the fermentation process, and its development has continued to grow rapidly in industries, particularly in terms of technological process and methodological innovation. Though, the challenges faced to achieve good lactic acid purity (99–100%) after the fermentation process are still a bottleneck for the industries to tackle. The lactic acid produced from fermentation broth usually contains numerous impurities such as bacterial cells, residual carbohydrates, proteins, vitamins, and phenolic compounds. Besides, by-products could also be produced throughout the fermentation period, which included alcohols (e.g., ethanol), glycerol, carbon dioxide, and organic acids (e.g., acetic acid or formic acid) (Ghaffar et al. 2014; Buyondo and Liu 2011). Furthermore, salt derivatives from lactic acid may also present in the fermentation broth due to alkaline reagents usage as a pH control (Kim and Moon 2001).

In addition, extensive studies have been circulated on a sustainable biorefinery concept where the biomass residue, especially lignocellulose materials, has been incorporated in the fermentation system as major carbon sources. This innovative methodology utilizing biomass (2G-biorefinery) had generated several kinds of bioproducts such as bioethanol, bioplastics, surfactants resin, biochemicals and a range of acids like citric and lactic acid (Ramos et al. 2017). Through this methodology, non-food raw material such as agricultural residues, energy crops, forestry and municipal solid wastes have been reused as a substrate for fermentation process. However, the bioproducts produced through this platform have more complex carbon and mixture of components than 1G-biorefinery products. 1G-feedstock normally uses simple sugar from food resources such as potato (glucose), maize (glucose), sugar cane (glucose and fructose), etc. While 1G-biorefinery is known as having food and feed that are for human consumption, the 2G-biorefinery can be derived from non-food material (Chowdhury et al. 2018).

According to Alvira et al. (2010), other form of impurities containing furans and phenolics compounds, such as furfural, hydroxylmethyl furfural (HMF), syringaldehyde and vanillin that may interfere with fermentation process could be derived from 2G lignocellulose feedstock (Bai et al. 2015). These impurities are mainly generated during the lignocellulose substrate's treatment phase. Thus, the cost to purify 2G-lactic acid is expensive and the process is complicated due to high interference of these impurities. This factor needs to be considered for lactic acid production to avoid difficulties during purification process.

Traditionally, the separation of lactic acid from fermentation broth was done through chemical precipitation, but the negative implication of gypsum generation on environmental issues (Seong et al. 2016) has urged the development of other alternative methodologies that can improve the process to a greener environment; where minimum chemical disposal, usage of fewer environmentally harmful or resource-depleting raw material and low direct manufacturing costs were targeted. Among the extractive fermentation techniques, adsorption by ion-exchange resin has been intensively studied and used for lactic acid separation and recovery from fermentation broth (Pradhan et al. 2017; Garrett et al. 2015; Rampai et al. 2016; Zhang et al. 2018). The strategy has been used in downstream processing of several other substances, especially for organic acids separation (e.g., acetic acid, citric acid, and succinic acid) from fermentation broth (Jianlong et al. 2000; Kurzrock and Weuster-Botz 2010; Karekar et al. 2020) as well as recovery of phenolic compounds (e.g., phenol and anthocyanins) and monosaccharide sugars from liquid solution (Chen et al. 2017, 2018; Trikas et al. 2017). The ion-exchange resin features provide several advantages that can elevate the lactic acid production to a higher level, alongside other benefits that can reduce the labor cost and time. The methodology has been improved with various kinds of modifications, and recently, the in-site separation by ion-exchange resin has been innovated for better lactic acid separation and recovery from fermentation broth (Othman et al. 2017b, 2018; Boonmee et al. 2016). Several important factors such as pH, temperature, type of resin, and eluent need to be considered to apply this methodology into the fermentation system. Other techniques of extractive fermentation have also been developed and applied various principles that may come with distinct advantages and disadvantages. The lactic acid recovery from fermentation broth may seem complicated and challenging; thus, multiple aspects were reviewed to imply ion-exchange resin's potential as a functional tool in high-value lactic acid production.

## Biotechnological concept of lactic acid production

Lactic acid (2-hydroxypropanoic acid) is the common industrial chemical known for various industrial applications in food, cosmetics, textile, pharmaceutical and has recently had emerged into the bioplastic industry for poly-lactic acid (PLA) (Abdel-Rahman and Sonomoto 2016). The feasibility of the dual-functional lactic acid groups (d- and l-lactic acid) makes it versatile for various chemical transformations and products (Biddy et al. 2016). It is recognized as “Generally Recognized as Safe” (GRAS) by the Food and Drug Administration (FDA) (Food and Drug Administration 2015). Lactic acid was also listed as a potential building block for future use in a United States Department of Energy Report (de Jong et al. 2012), making it one of the most useful chemicals that received a great deal of attention worldwide. Primary producers and lactic acid consumers are primarily dominated by the United States, Western Europe, and the Asia-Pacific region. The largest lactic acid producer is PURAC®, which accumulated about 45% of lactic acid world production, with an installed capacity of approximately 350,000 tons per year (Hörhammer et al. 2014). The global lactic acid market size value was at USD 3.7 billion in 2020, with revenue forecast in 2025 was estimated at USD 8.7 billion. The lactic acid industry's growth rate was expected at a compound annual growth rate (CAGR) of 18.7% from 2019 to 2025 (Grand Review Research 2019). One of the growing applications of lactic acid is as a monomer for biodegradable polymer poly-lactic acid (PLA). The presence of a chiral carbon atom in the lactic acid structure, which is optically active, supports the formation of the crystalline and PLA stability (Kumar et al. 2019). PLA also exhibits high heat deflection and good biodegradability, naturally. It is environmentally friendly and has a high potential to replace conventional petrochemical plastic material (Pal and Dey 2012). Presently, the world market demand for PLA is nearly 10^5^ metric tons per year and was predicted to rise to 28% in 2025, due to the application of PLA as an good substitute for polyethylene terephthalate (PET) (Daful et al. 2016).

Furthermore, fermentation substrates for 2G-lactic acid production using renewable low-cost materials have recently emerged, as it does not compete with the food and could reduce the environmental problems. The biotechnological process for 2G-lactic acid production using unutilized feedstock waste such as corn stover (Garrett et al. 2015), dried distiller’s grains solubles (Zaini et al. 2019a, b), cassava bagasse (Yuwono et al. 2017), etc., have become great alternative approaches in the industry. The shift to utilizing feedstock waste led to the minimized production cost and maximized renewable biomass utilization during the upstream process. However, the production of 2G-lactic acid from feedstock waste requires efficient downstream processing to remove impurities. This stage is one of the most critical bottlenecks in industrial lactic acid production (Cubas‐Cano et al. 2018). The primary importance of downstream processing is to provide a high degree of lactic acid purity with cost-effective production, as this stage may take up to 30–40% of production cost (López-Garzón and Straathof 2014). The challenge in achieving high 2G-lactic acid purity is due to lack of economic methods to completely remove any biomass residues and fermentation process impurities (Bernardo et al. 2016; Järvinen et al. 2000).

The cellulosic biopolymers of biomass are made up of sugar ring chains that are linked with strong hydrogen bonds where, these bonds are not present in the typical starch structure of 1G-feedstock. When this complex structure of lignocellulosic biomass is broken down into smaller components, the by-products produced will become impurities and interfere with fermentation process efficiency. According to Malav et al. (2017), furfural and HMF formed during hydrolysis of biomass may become part of potent fermentation inhibitors which results in poor fermentability of biomass hydrolysate. Thus, the overall production of 2G-lactic acid is expected to be more expensive and challenging compared to 1G-lactic acid. Particularly, the separation and recovery of 2G-lactic acid from fermentation broth requires several steps and energy intensive to achieve high lactic acid purity. Practical approaches using ion exchange adsorption resin is targeted on improving the efficiency of lactic acid separation and recovery in 2G-fermentations system. This plays a great importance to produce a higher yield of lactic acid at a lower cost.

The production of lactic acid through the fermentation of lactic acid bacteria (LAB) is the industry method due to ecological factors and the emerging world’s technologies. The process requires low energy consumption, low production temperature, and capable of producing pure lactic acid of specific stereoisomer, i.e., l-( +)-lactic acid or d-( −)-lactic acid when specific strains were used (Juodeikiene et al. 2015; Komesu et al. 2017b). Approximately 90% of total lactic acid produced worldwide is carried out through bacterial fermentation, as chemical synthesis has a significant disadvantage of producing a racemic mixture of lactic acid (Dumbrepatil et al. 2008). Pure fermentation substrate, which includes carbon (e.g., glucose and sucrose) and nitrogen sources (e.g., yeast extract or peptone), is required for high production yields. However, these refined nutrients come at a high price, which would increase the cost of production (Vandenberghe et al. 2018). Recent use of renewable and low-cost materials has provided advantages to further reduce overall cost (Zaini et al. 2019a; Yuwono et al. 2017). Apart from that, the high concentration of product generated (lactic acid) can also reduce the fermentation efficiency, especially in the batch fermentation process (Yuwono et al. 2008; Serrazanetti et al. 2013; Othman et al. 2017a). pH significantly affects the fermentation process, especially in batch mode due to the accumulation of lactic acid in the fermentation media. Generally, the culture broth's pH should be maintained in the range of 5 to 7 for optimum lactic acid production. However, as lactic acid is the primary metabolic end-product, the medium's pH will significantly drop as the fermentation progresses and subsequently affect bacterial growth (Abdel-Rahman et al. 2013; Komesu et al. 2017b). Chahal (1990) and Litchfield (1996) reported that bacteria's growth is likely to stop when the levels of free lactic acid reached 1–2 wt % of total combined lactic acid. LAB fermentation without any neutralizing or buffering agents will decrease pH during bioconversion due to the dissociation of acid, which eventually leads to slow fermentation kinetics, inhibiting the bacterial metabolism, and reducing lactic acid yield (Moldes et al. 2003).

However, recent studies on in-site separation of lactic acid by ion-exchange resin had maintained the pH during fermentation process (Boonmee et al. 2016; Othman et al. 2017a, b; Othman et al. 2018). The ion-exchange resin in fermentation media quickly adsorbs lactic acid produced by LAB to avoid growth limitation by low pH. The approach had a foresight to develop a better lactic acid recovery technique from fermentation broth as well as to be incorporated in lactic acid production system in industry.

## Ion exchange adsorption as alternative way for lactic acid recovery

Lately, the adsorption strategy using ion-exchange resin is getting much attention from researchers as it was found to be one of the most effective methods for lactic acid recovery. It implies the approach of neutralizing fermentation broth by removing lactic acid selectively in-site, resulting in a low level of lactic acid in the fermentation broth (Wang et al. 2014; Boonmee et al. 2016; Othman et al. 2017a). The risk of product inhibition is reduced, and no precipitate (gypsum) is produced in the extractive method.

The application of adsorption technology, especially by ion-exchange resin, has been extensively used in various areas such as water purification (Tabassum 2019), wastewater treatment (Batubara et al. 2019), and desalination of seawater (Seong et al. 2016). It is also applied widely in food research development, particularly in the de-acidification process of *noni* juice (Haslaniza et al. 2015) and starfruit (Fong et al. 2017), as well as facilitate the reduction of saponin bitterness in *Carica papaya* leaf (Syed Amran et al. 2018) and the deodorization of fucoidan from brown seaweed (*Sargassum sp*.) (Khalafu et al. 2017). However, this review will focus on applying ion exchange adsorption onto lactic acid separation and recovery from the fermentation process.

### Ion exchange adsorption process of lactic acid

Adsorption is a well-established technique using adsorbents to recover lactic acid from fermentation broth. Theoretically, this includes the deposition of liquid phase components (adsorbate) onto the solid phase (adsorbent) surface, which may often be explained by electrical attraction to the solid surface components with a low electrical charge that will eventually form a monolayer of the molecular, ionic, or atomic film (Ruthven 1984; Okeola and Odebunmi 2010). Regularly, an equilibrium concentration is rapidly formed on the surface and may occasionally be followed by slow diffusion into the adsorbent particles (Jørgensen 1989). The accumulation of molecules on the adsorbent surface is influenced by various interactions such as hydrophobic, electrostatic attraction, and hydrogen bonding (Koźlecki et al. 1997; Yang et al. 2007). The adsorbent surface has unique electronic and steric properties of matrix configuration, which caused varied energy levels based on the degree of association with the adsorptive surface (Kammerer et al. 2010). The commonly used adsorbents are natural materials, metal-based adsorbents, sewage sludge, industrial waste materials, industrial by-product clay, nanomaterials, porous carbon, and silica (Abdulaziz and Musayev 2017; Kulkarni et al. 2018; Garba et al. 2019). Different types of adsorbents may possess other properties and advantages. For example, polyvinyl pyridine adsorbents are mainly used to adsorb lower carboxylic acids and easily desorbed with alcohol eluent; however, it is challenging to separate lactic acid from alcohol (Xu et al. 2018; Uslu et al. 2016). Meanwhile, silicate pellet adsorbent can separate lactic acid and eliminate more significant components, especially protein. Besides, it has good thermal and hydrothermal stability, despite its very poor water affinity (Aljundi et al. 2005).

Ion exchange involves the exchange of ions between a liquid and a solid phase. This process requires low-cost and straightforward equipment, but is typically applied at a low salt concentration (Kumar et al. 2019). The chemical energy at equilibrium after the ion exchange process is lower than before the process is started. Theoretically, the ratio in the numbers of ions released is equivalent to the number of ions taken up in a completely pure ion exchange process, which is hardly observed. A mixture of two processes of adsorption and ion exchange is most often observed in nature. Therefore, the adsorption and ion exchange processes are considered together, as they share several common features, especially in the design concept. However, in some cases, their operating cycles may be similar, although ion exchange operating cycles are slightly more complex. Ion exchange can be categorized into anion and cation exchange based on the ionic group attached to the resin (solid). Each category can be further subdivided based on their charge into weak acid or strong acid exchanger, which will have a different structure, functional groups, and polymer matrix (Evangelista and Nikolov 1996). Cation resins have negatively charged groups fixed to the backbone material that only allow cations to pass through and reject anion. It releases positive ions (e.g., H^+^ or Na^+^) in exchange for impurity cations present in the broth. Meanwhile, anion resins have positively charged groups fixed to the backbone material that only accept anions to pass through and reject cations. It releases negative ions (e.g., OH^−^ or Cl^−^) in exchange for impure anions present in the broth (Luca 2000).

Anion exchange resin is frequently used in the separation and purification of lactic acid from fermentation broth. As lactic acid exists in the form of anionic lactate ions (C_3_H_5_O_3_^−^), it needs to bind to a cationic molecule for separation. Several commercial anion-exchange resins have been previously used for separating lactic acid from the fermentation broth, including Amberlite IRA-67, IRA-96, IRA-92, IRA-400, Lewatit S3428, DOWEX-XUS 40,196, and DOWEX-50) (Evangelista and Nikolov 1996; Tong et al. 2004; Quintero et al. 2012). However, different resin types may have different lactic acid adsorption selectivity and capacity that can be further determined using several adsorption isotherms models such as Langmuir, Freundlich, Langmuir–Freundlich, and many more (Zaini et al. 2019b; Othman et al. 2017b). Apart from that, there is another step involved in the ion exchange equipment, where cations or anions from the fluid deposit in the resin and displace equivalent amounts of other ions from the resin. The step will replace all ions in the resin, but the activity can be restored by allowing the exhausted resin to be in contact with a high concentration of the desired ions, for example, a strong acid to replace lost hydrogen ions (Couper et al. 2012).

The separation of lactic acid from the fermentation broth by ion-exchange resin occurred during the downstream processing. Several researchers have developed practical and cost-effective downstream processing routes for lactic acid separation and recovery. Quintero et al. (2012) indicated that the process of lactic acid separation by ion-exchange resin might involve three stages: 1) lactate ion adsorption; 2) elution; and 3) lactate conversion into lactic acid. Generally, the lactic acid is separated, recovered, and purified through several ion-exchange adsorption operation processes. Nonetheless, it is almost possible to get a pure lactic acid crystalline form because of its high affinity for water and the tendency to form lactate dimer at a concentrated state (Lunelli et al. 2010). Besides, the fermentation broth may have a diluted lactic acid solution, mixed with other impurities that formed throughout the fermentation process, making the process more complicated. Bernardo et al. (2016) stated that the presence of lactate salts and other impurities from the fermentation process during lactic acid extraction might become a significant drawback in the downstream processing stage.

**Fig. 1:**
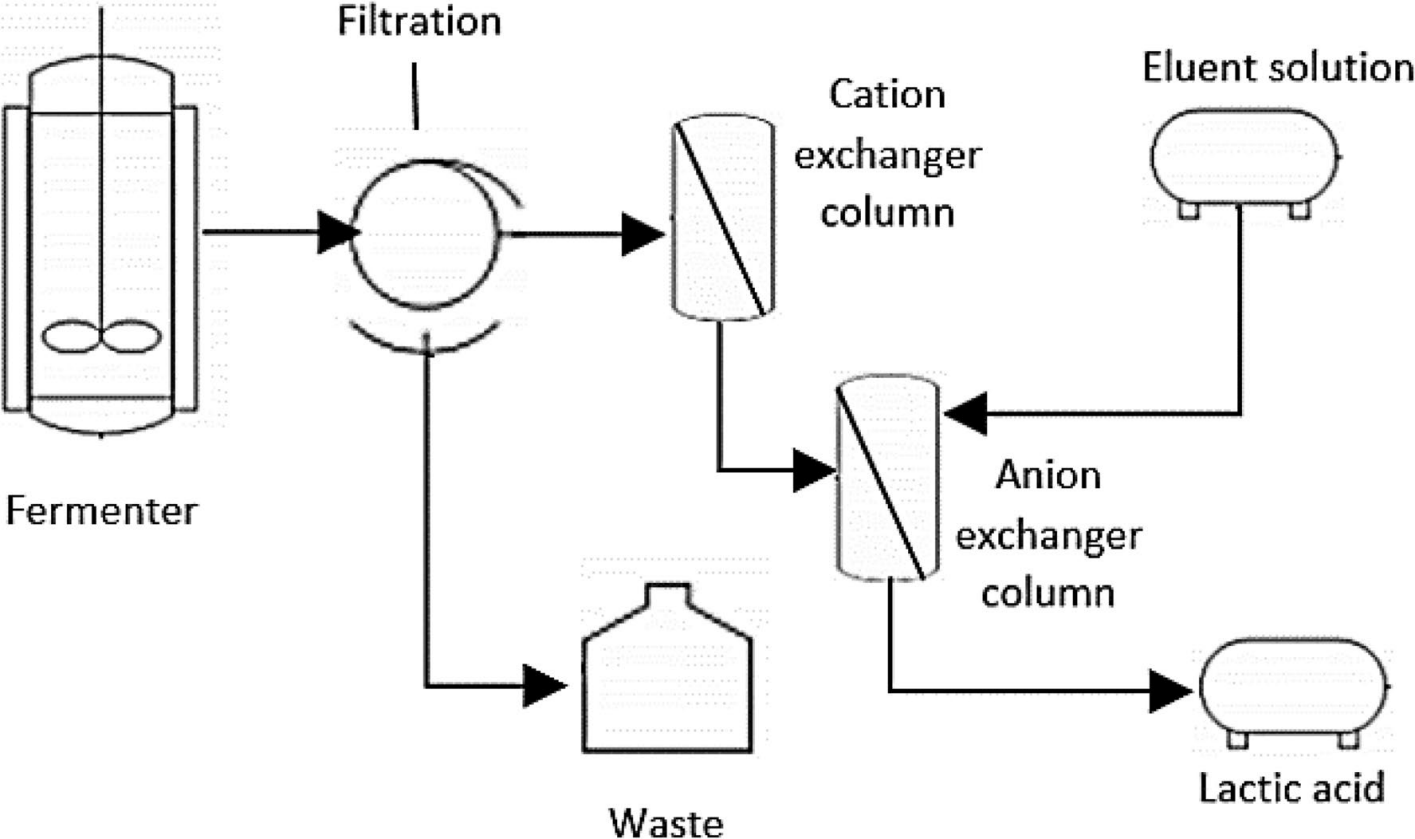
Schematic diagram of extractive ion exchange lactic acid process

The common operation process for lactic acid separation started with lactic acid production by the fermentation process in the fermenter and proceeded to the filtration process to avoid contamination by microorganisms that might enter the ion exchange column. Generally, the fermenter is connected to two columns packed with an ion-exchange resin. The addition of sodium hydroxide to the fermentation process generates sodium lactate. The first column of the cation exchanger is meant to acidify the fermentation broth, aiming to convert sodium lactate into lactic acid as sodium ions are exchanged with hydrogen ions. No eluent is needed at this stage, as lactic acid does not bind to the cation exchanger. When the pH of effluent increases, the resin is considered saturated with Na^+^ ion, and all undissociated lactic acid will pass through the column. Considering that resins (anion exchange) absorb the undissociated lactic acid form more effectively compared to the dissociated state, broth acidification is required before elution through the resin. After that, the acidified broth containing undissociated lactic acid is passed through an anion-exchange resin to separate the lactic acid molecule from the fermentation broth. Elution by liquid adsorption took place at this stage as lactic acid is recovered through the eluent. This extractive operation process of lactic acid separation and recovery has been reported in several studies (Zaini et al. 2019b; Evangelista and Nikolov 1996; González et al. 2006). The schematic diagram of the extractive lactic acid process is illustrated in Fig. 1.

**Fig. 2:**
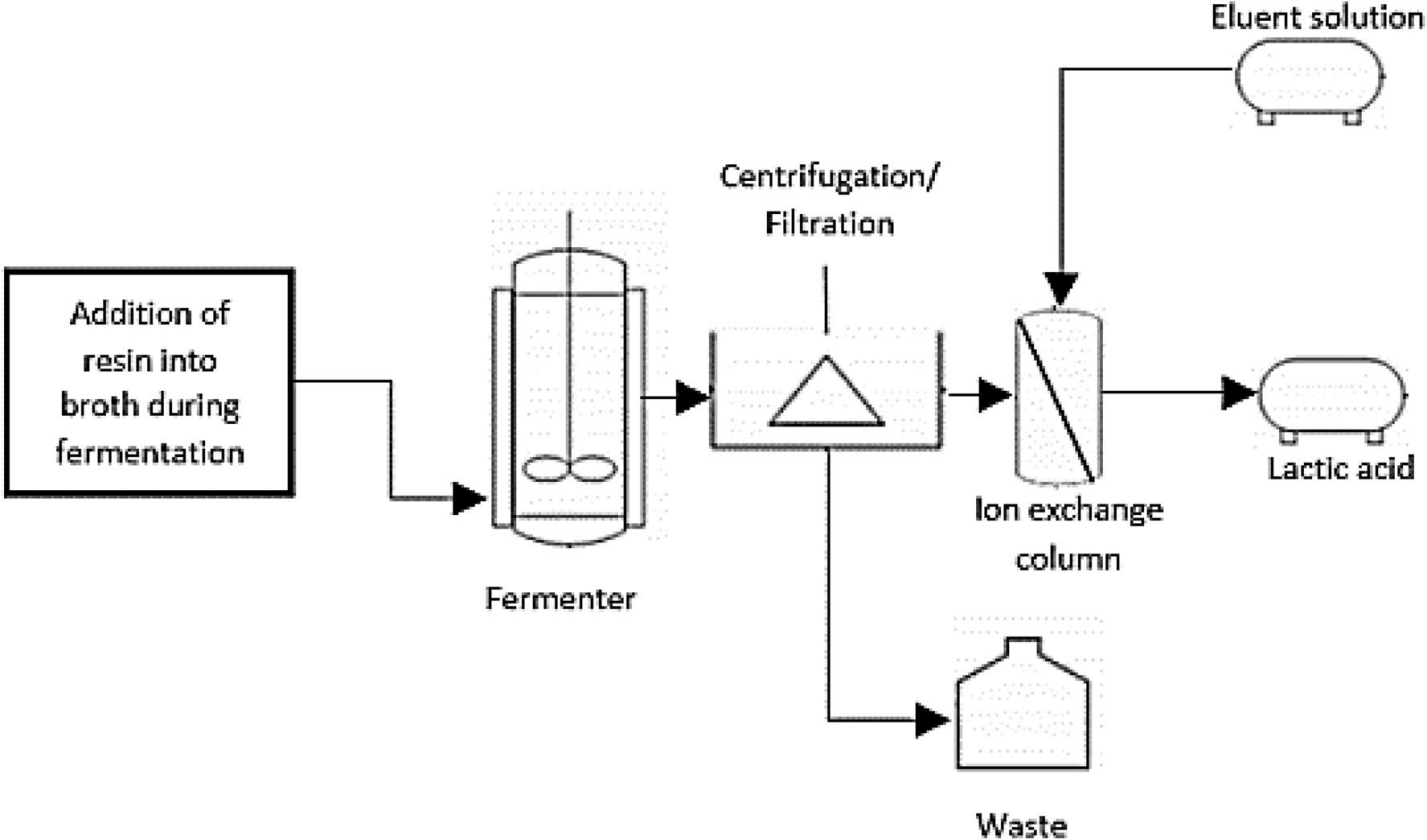
In-site ion exchange separation of lactic acid from fermentation process

Apart from that, an alternative process operation was studied. The resin is directly added to the broth to improve further the in-site removal of lactic acid from the fermentation process. This technique was recently discovered by Boonmee et al. (2016) and Othman et al. (2018). Figure 2 illustrates the basic layout of the overall process operation.

In this operation, a more practical approach with minimal and simple process control equipment was used. The resin was added to simultaneously maintain the pH of fermentation broth, without any addition of alkali as the pH regulator during the fermentation process. Usually, weak anion-exchange resins are used due to their properties that could adsorb lactic acid below and above its acid dissociation constant. This fact is advantageous, as it does not require fermentation broth acidification before adsorption (John et al. 2008). The lactate ion is adsorbed onto resin during the lactic acid fermentation, neutralizing the fermentation broth's pH and eliminating the end-product inhibition throughout the process. Upon completion of lactic acid adsorption onto the resin, the centrifugation or filtration process may separate resins from the remaining broth (waste). However, traces of lactic acid could be present in the waste depending on the lactic acid ion-exchange adsorption process's efficiency. Simultaneously, the elution process of recovered resin packed in the column is operated, eventually producing lactic acid. The formation of salt did not occur in this developed process as no alkali is present to convert the lactate ion to sodium lactate. After the final elution step, the saturated and exhausted ion-exchange resin will be regenerated and reused in the following ion adsorption processes for economic reasons. Commonly, the regeneration process of resin is performed alternately and intermittently. The step typically employed NaOH as regeneration eluent and washing with water to remove the eluent’s excess from resin. Simultaneously, the operational cost is reduced, and less waste is produced at the end of the process (Boonmee et al. 2016). The process is relatively more straightforward and has shorter downstream processing routes compared to the previous ion exchange operation process. Idler et al. (2015) stated that numerous downstream processing steps significantly affect the product's quality and price.

#### Lactic acid separation by ion exchange adsorption mechanism

The ion exchange adsorption mechanism is a heterogeneous chemical process that involves ion transfer to and from the interphase boundary. In other words, it is a process of removing the ions from an aqueous solution and replacing it with another ionic species that are bound at the solid phase via electrostatic interactions to achieve electroneutrality. The ion exchange process occurs in two ionic flux forms, either into the ion exchange particles or in the opposite direction out of the ion exchange particles (Kammerer et al. 2010).

**Fig. 3:**
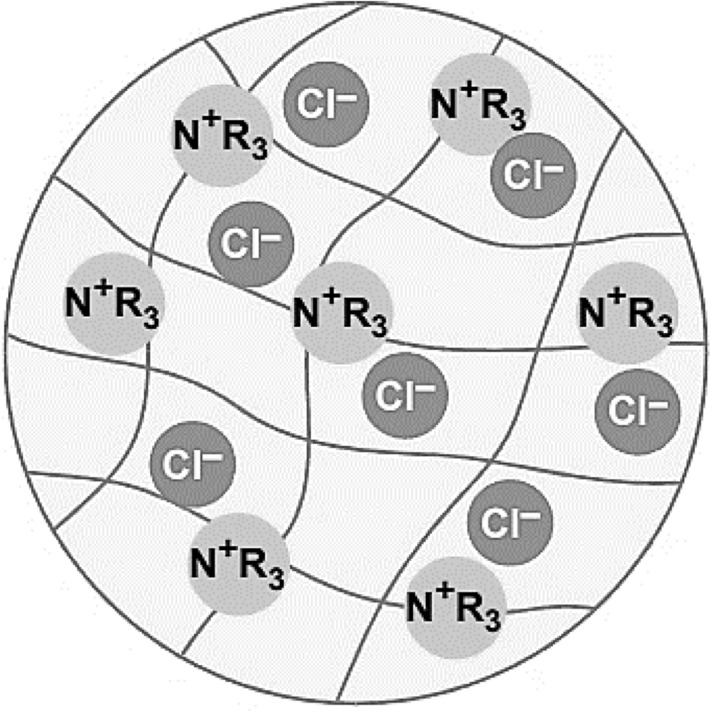
Schematic anion-exchange resin beads. By permission from Rohm and Haas, Inc., Pennsylvania, United States (Rohm and Haas 2008)

The diffusion inside the absorbent material and diffusion in the surrounding aqueous solution has been considered the major phase in the ion exchange process. Throughout the process, the formation of a thin film on the absorbent's surface is unavoidable and considered the intermediate phase. This interphase film cannot be removed, and rigorous agitation can only reduce the film's thickness. The transportation of existing ions in the aqueous solution into the exchanger, replacing the counterion that diffuses out of the exchanger crossing the film, is the phenomenon known as ion exchange. The counterions are exchangeable ions present in the exchangers that move freely within the framework. However, their movement must be fulfilled by the electroneutrality principle, which means that any counterions that leave the ion exchanger need to be replaced with another counterion that has the equivalent quantity to the previous one. This counterions movement may either occur in the opposite direction (ion exchange) or by existing as co-ions in the same reaction (Kumar and Jain 2013). Meanwhile, a co-ion is a fixed ion that has been permanently attached to an ion exchanger. It is immobile, insoluble, and a part of the structure. The overall illustration of an ion exchanger (resin) is shown in Fig. 3. The skeleton of anion resin used to separate lactic acid has dark lines representing the polymeric skeleton of resin beads that are porous and contain water. The co-ion functional groups illustrated are quaternary ammonium cations (CH_2_^−^N^+^-(CH_3_)_3_), simplified as N^+^R_3_ attached to the skeleton. The resin bead's counterion is chloride anions (Cl^−^), which is the standard delivery form for many anion resins (Rohm and Haas 2008). The counterion of Cl^−^ will exchange with lactate ions (C_3_H_5_O_3_^−^) in the fermentation broth and eventually preserve the electrical neutrality of the resin with the present co-ion.

**Fig. 4:**
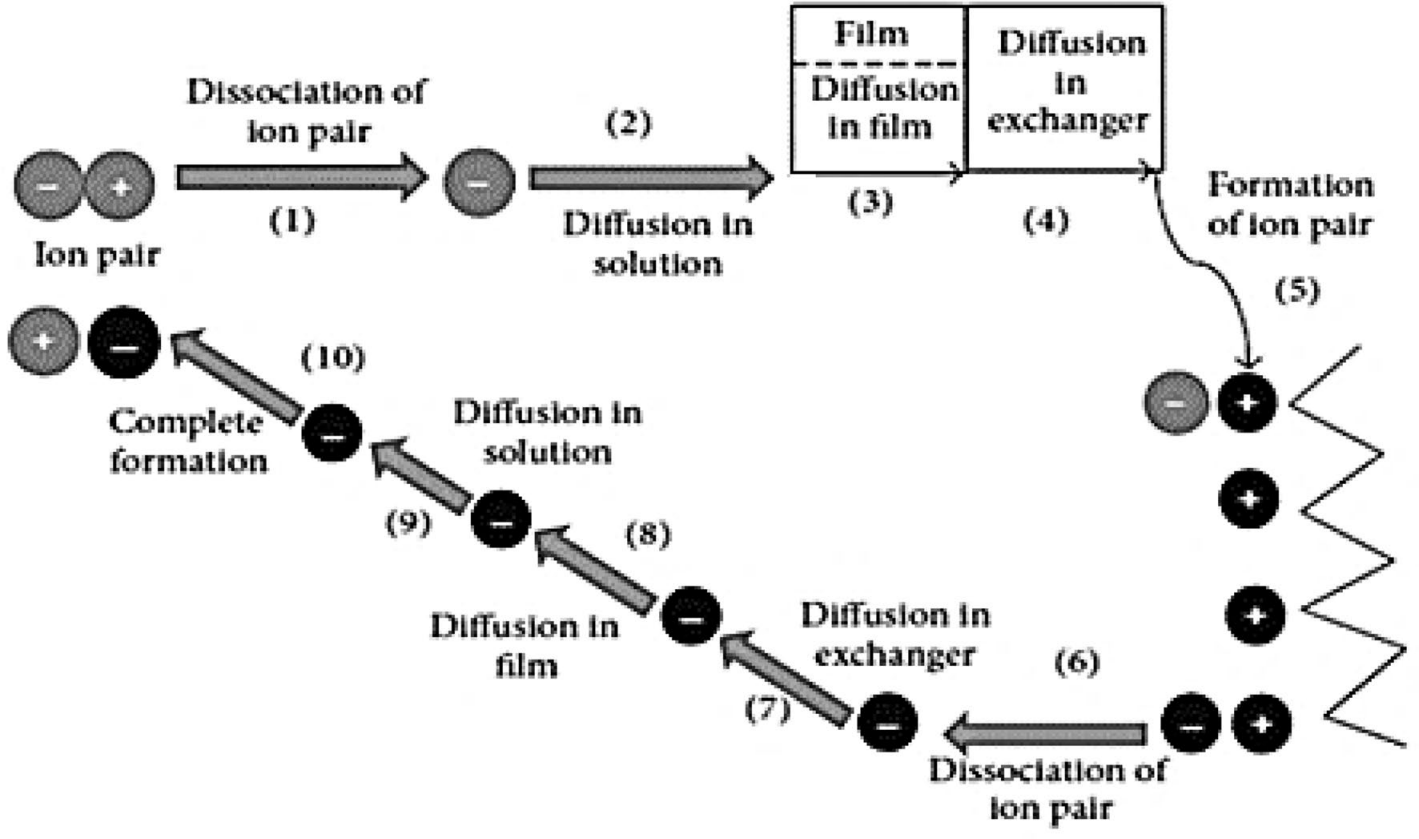
The general step of ion exchange processes. Modified from Kumar and Jain (2013) with permission from Hindawi

Generally, the ion exchange process for lactic acid (C_3_H_6_O_3_) involves the following steps: 1) dissociation of the lactic acid compound into lactate ions (C_3_H_5_O_3_^−^) and hydrogen ions (H^+^) in the liquid; 2) the diffusion of lactate ions from the liquid phase towards the interphase film of resin; 3) the diffusion of lactate ions out of the film, and 4) into the material phase step of the resin. The material phase step includes the formation of different ion pairs, where 5) lactate ions associate with other functional groups bound to the resin, and 6) the dissociation of the primary ion pair of functional groups that have been replaced and removed by lactate ions, 7) before diffusing inside the material phase. Subsequently, the removed ions move to the surface step, which 8) diffuses into the interphase film, followed by 9) the diffusion into the liquid phase, where random distribution occurred. Finally, 10) the formation of removed ions with hydrogen ions in liquid takes place. The overall general step is illustrated in Fig. 4.

#### Lactic acid recovery by liquid adsorption and further purification

**Fig. 5:**
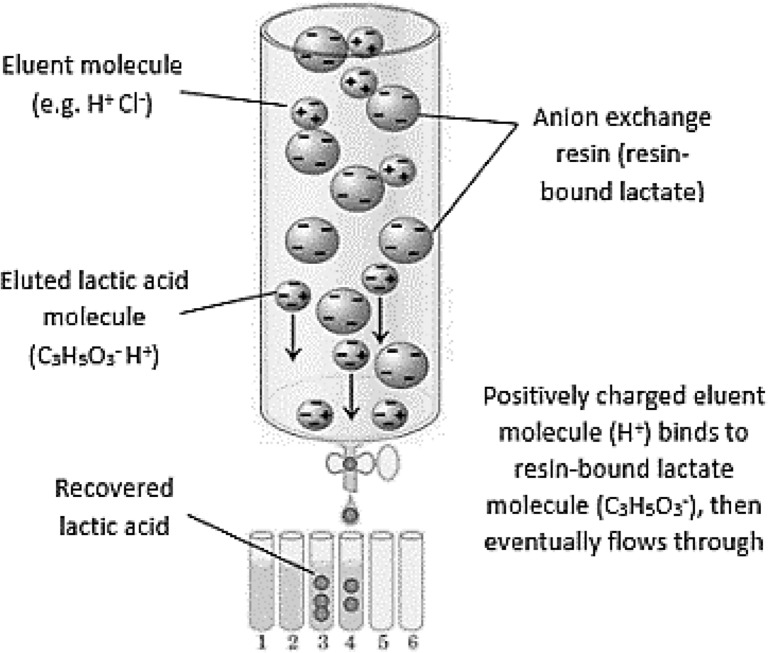
Liquid adsorption/elution process to recover lactic acid. Modified with permission from Bio-Resource (Bio-Resource 2011)

At the end of the ion exchange process, the recovery of lactic acid bound to resin is made by liquid adsorption, known as the elution or desorption step. A specific solvent is used as the eluent to desorb all resin-bound lactate, eventually recovered as lactic acid in the final eluate. For example, hydrogen ion (H^+^) in HCl is capable of desorbing the bound lactate ion (C_3_H_5_O_3_^−^), which eventually caused the binding of chloride ion (Cl^−^) onto the resin, replacing the lactate. Finally, the formation of undissociated lactic acid is eluted and recovered (Fig. 5) (Bio-Resource 2011).

Generally, the efficiency of elution and recovery of lactic acid is influenced by several factors that might also affect its purity. Elution conditions such as flow rates and eluent concentrations are among the significant factors. These factors are essential to ensure that all lactate-bound resin is detached and recovered as lactic acid. Though, an insignificant effect of the eluent flow rates was observed by Boonmee et al. (2016), where the factor did not influence the amount of lactate eluted, resulting in the larger consumption of eluent volume by the higher eluent flow rates. The study also reported a minor effect of eluent concentrations, where high concentration does not contribute to the reduction of eluent volume used to recover the lactic acid. Consequently, the study used 1.0 M HCl at a low flow rate of only 0.1 BV/min, which resulted in 80% lactic acid recovery compared to resin-bound lactate. However, Zaini et al. (2019b) reported the yield of 96 and 100% of lactic acid recovery at 0.5 and 1.0 M concentrations of HCl. Meanwhile, HCl concentrations below 0.5 M (~ 0.05 M to 0.1 M) have low efficiency in recovering the lactic acid. It shows that eluent strength plays a critical role in lactic acid desorption and detachment from the resin. Yet, due to the economic factor, the 0.5 M HCl concentration was selected with a total lactic acid recovery of ~ 80.40% after the elution (3 mL/min flow rate). Furthermore, increasing the HCl concentration or strength could decrease the percentage of lactic acid purity (Bishai et al. 2015). Apart from that, several studies reported using other types of lactic acid eluent, such as methanol, NaOH, and H_2_SO_4_, with varied recovery efficiency depending on the concentration and process flow rates (Luongo et al. 2019; Delgado et al. 2018; Pleissner et al. 2017).

Nevertheless, the recovered lactic acid could be present at low concentration and purity, as impurities such as eluent components and organic molecules are present in the final lactic acid eluate. Thus, additional steps could be added after lactic acid purification by ion exchange, such as filtration, electrodialysis, and vacuum distillation (Neu et al. 2016). Further purification process such as evaporation or distillation may increase the purity of lactic acid and provide the advantage to regenerate the desorbent eluent from the extract and raffinate streams to be reused in the process. Kulprathipanja and Oroskar (1991) proposed that the eluent preferably has a substantially different average boiling point (higher or lower than 5 °C) than the feed mixture, so the separation of at least a portion of desorbent eluent by fractional distillation is possible. Earlier, Evangelista and Nikolov (1996) had successfully incorporated an evaporator to recover the methanol eluent from the extract and raffinate streams during the desorption of lactic acid from anion-exchange resin. Recently, Delgado et al. (2018) reported the recovery of methanol from the extract and raffinate by waste-heat driven distillation and simultaneously resulted in the high level of final lactic acid purity (99.76%). Another lactic acid purification was done by Neu et al. (2016), which incorporated bipolar electrodialysis membranes and distillation into the ion exchange adsorption process of lactic acid, eventually leading to an improved lactic acid optical purity (99.8%). Technically, the lactic acid produced through these processes is recovered at a concentrated state. However, several studies reported pure lactic acid production in the diluted eluate, which is directly collected after the elution step. This finding highlights that the lactic acid concentration in the eluate is not directly proportional to lactic acid's purity (Bishai et al. 2015). For example, a study by Zaini et al. (2019b) has reported a high lactic acid purity (91.8%) in the diluted eluate of lactic acid where the overall 2G-lactic acid recovery and purification steps include treatment with 7% (w/v) activated carbon and ion exchange adsorption system, comprising the cation and anion-exchange column. Similarly, Bernardo et al. (2016) purified the broth with powdered activated carbon (18.72%), and the yield was nearly 100% pure 2G-lactic acid. Another production of a highly pure 2G-lactic acid (99.17%) was observed in the study by Bishai et al. (2015) using a simple two-step purification process of anion and cation exchange resin without the incorporation of activated carbon during the purification step.

### Reported research of ion exchange adsorption

Lactic acid recovery using ion-exchange resin has been utilized and intensively studied by many researchers for the past years. Ion-exchange resin has been proven as an important tool to recover and produce lactic acid with better productivity, purity, and yield. Studies of lactic acid recovery using this method on 1G and 2G feedstocks are summarized in Table 1.

**Table 1:** Fermentation studies using ion-exchange resin for lactic acid recovery

Ion-exchange resin	Microorganism	Substrate	Product class^a^	Lactic acid				Biomass production	References
				Adsorption	Production (g/L)/productivity (g/Lh)	Recovery yield	Purity		
Amberlite IRA-67	*Lactobacillus delbrueckii* NRRL-B445	Processed Eucalyptus wood	2G	0.273 g lactic acid/g dry resin	21 g/L	99.0%	–-	–	(Moldes et al. 2003)
	*Lactobacillus. lactis* ATCC 11,454	Lactose	1G	0.15 g lactic acid/g resin	5.9-fold enhancement compared to fermentation without resin	70%	–	–	(Boonmee et al. 2016)
	*Pediococcus acidilactici*	Glucose	1G	0.996 g lactic acid/g wet resin	0.59 g/Lh	1.23 g/g	–	6.7 × 10^12^ CFU/g	(Othman et al. 2018)
	*Streptococcus equinus*	Jackfruit seed powder	2G	–	109 g/L	62%	–	–	(Nair et al. 2016)
	*Bacillus coagulans*	Corn stover	2G	112.2 mg lactic acid/g resin	0.329 g/Lh (1.31-fold enhancement compared to fermentation without resin)	0.90 g/g	–	–	(Garrett et al. 2015)
	*Lactobacillus coryniformis subsp. torquens*	Dried distiller’s grains solubles	2G	136.11 mg lactic acid/g of resin	–	80.4%	91.8%	–	(Zaini et al. 2019b)
	*Lactobacillus delbrueckii* NCIM 2025	Cassava bagasse	2G	126 mg lactic acid/g of resin	~ 17 g/L	95%	–	–	(John et al. 2008)
Amberlite IRA-67; IRA-400	*Lactobacillus rhamnosus* B103	Cheese whey	2G	–	143.7 g/L	13.40%	~ 100%	10.37 g/L	(Bernardo et al. 2016)
Amberlite IRA-400	*Lactobacillus* strain ATCC 10,863	Glucose	1G	pH = 2, 106 mg/g wet resin pH = 5, 97.09 mg/g wet resin	-	pH = 2, 92.11% pH = 5, 86.21%	–	–	(Cao et al.^2002^)
	*Lactobacillus delbrueckii* NRRL-B445	Sucrose	1G	0.923 g lactic acid/g resin	1.665 g/Lh (5.32-fold enhancement compared to fermentation without resin)	0.929 g/g	−	–	(Srivastava et al. 1992)
	*Bacillus sp* BC-001	Glucose	1G	1.8 g lactic acid/g wet resin	> 4 g/L	–	–	–	(Rampai et al. 2016)
	*Lactobacillus casei*	Whey	2G	–	0.984 g/Lh (fivefold enhancement compared to fermentation without resin)	0.85 g/g	–	–	(Ataei and Vasheghani-Farahani 2008)
	*Lactobacillus brevis*	Cassava flour	2G	0.109 g lactic acid/g resin	0.20 g/L/h	92.7%	–	–	(Quintero et al. 2012)
Amberlite IRA-96	*Lactobacillus amylophilus* GV6	*Zizyphus oenophlia*	2G	210.46 mg lactic acid/g resin	–	98.9%	99.17%	–	(Bishai et al. 2015)
	*Lactobacillus rhamnosus*	Apple pomace	2G	0.381 g lactic acid/g resin	30 g/L	102.1%	–	–	(Gullón et al. 2010)
Amberlite IRA-120; IRA-420	*Lactobacillus delbrueckii* CECT 286	Beet molasses	2G	–	> 60 g/L	0.91 g/g	–	–	(Monteagudo and Aldavero 1999)
Amberlite IRA-92	*Lactobacillus rhamnosus* ATCC 10,863	Paper sludge	2G	1.16 g lactic acid/g resin	–	82.6%	96.2%	–	(Tong et al. 2004)
Anion exchange resin 335	*Bacillus coagulans* CC17	Glucose	1G	402 mg lactic acid/g wet resin	0.96 g/Lh	82%	–	12.3 OD	(Zhang et al. 2018)
Anion exchange D319	*Lactobacillus plantarum* CMCC 8610	Glucose	1G	–	–	–	-	34.5 g/L (2.3-fold enhancement compared to fermentation without resin)	(Cui et al. 2016)
WA 30 ion-exchange resin	*Streptococcus bovis*	Cassava bagasse	2G	100 mg lactic acid/g resin	–	60%	80%	–	(Yuwono et al. 2017)
Amberlite FPA 53; CR 5550	*Bacillus coagulans*	Saccharose	1G	–	92.5 g/L	> 90%	–	–	(Pleissner et al. 2017)
Resin D301	*Lactobacillus casei*	Glucose	1G	–	0.482 g/Lh (1.47- enhancement compared to fermentation without resin)	0.788 g/g	–	–	(Jianlong et al. 1994)
Lewatit S2568H; Lewatit S3428	*Lactobacillus helveticus*	Sweet whey	2G	–	32.7 g/L	–	> 99.9%	–	(González et al. 2006)
RELITE EXC 08; RELITE EXA 133	*Bacillus coagulans*	Coffee mucilage	2G	–	4–5 g/L/h	0.70–0.77 g/g	99.8%	–	(Neu et al. 2016)
	*Bacillus coagulans* A-35	Sweet sorghum juice	2G	–	1.77 g/L/h	0.78 g/g	98.9%	–	(Olszewska-Widdrat et al. 2019)

^a^*1G* first generation feedstock, * 2G* second-generation feedstock

Various parameters and factors involved in lactic acid recovery were previously investigated to enhance the final result. According to Gao et al. (2010), resins need to possess high capacity and selectivity for lactic acid over water and substrates and show good regenerability and biocompatibility with microorganisms for successful lactic acid removal. Several factors that may influence the resin adsorption capacity and efficiency are absorbent properties (porosity, surface area, particle size, and functional group), the absorbate (polarity, pKa, molecular weight, structure, and solute concentration), and also conditions of the process (pH, temperature, contact time, and mixing speed) (Yousuf et al. 2016). Therefore, many previous studies have been conducted to determine the factors or to find the optimum condition for lactic acid recovery from fermentation broth.

#### Effect of pH on lactic acid adsorption

One of the crucial factors to control lactic acid separation and increase lactic acid adsorption is pH. Kulprathipanja and Oroskar (1991) patented the method for 1G-lactic acid separation and recovery from the culture broth of *Lactobacillus delbrueckii*, *Lactobacillus bulgaricus*, and *Lactobacillus leichmannii* using anion polymer adsorbents. The authors compared strong, moderate, and weak base anion-exchange resins and found that lactic acid's best adsorption efficiency occurs below its pKa value (3.86). A similar study by Evangelista et al. (1994) compared several anion base resins such as weak (VI-15 and Reillex 425), moderate (MWA-1, WGR-2, and XUS 40,283), and strong (XUS 40,196) to evaluate the lactic acid adsorption capacities at different pH. The result showed that lactic acid adsorption of weak and moderate base resin was good when the pH value of fermentation broth below the pKa. However, several researchers reported that weak base resin's adsorption onto lactic acid may also occur above their pKa value. For example, Tong et al. (2004) purified 2G-lactic acid using a weak anion-exchange resin (Amberlite IRA-92) and study showed that the increased pH from 5 to 6 had a positive effect on 2G-lactic acid purity (96.2%), yield (82.6%), and productivity (1.16 g lactic acid/g of resin). Furthermore, Moldes et al. (2003) also successfully recovered 2G-lactic acid at the pH of 4.4 using weak base resin (Amberlite IRA-67); where the study achieved more than 99% lactic acid recovery.

Meanwhile, for strong base resin, the lactic acid adsorption was stable in a broader pH range of 2 to 6, but strong eluents were required to regenerate this resin. Thus, cation exchange resin was commonly used to acidify the fermentation broth before being passed to weak and moderate anion-exchange resin. About 6.8-bed volume (BV) of methanol can completely recover all absorbed lactic acid from the strong base exchange resin with high purity (99%) (Evangelista and Nikolov 1996). In the study by González et al. (2006), 2G-lactic acid was purified from the whey fermentation broth of *Lactobacillus helveticus* using Lewatit S2568H and Lewatit S3428. The strong cation resin, Lewatit S256H, was used in the first step to acidify the fermentation broth (pH < 6) then, weak anion resin, Lewatit S3428, was used for lactic acid purification. Towards the end, more than 99% purity of 2G-lactic acid was produced as the final product, with production value of 32.7 g/L. Another type of strong base resin (Amberlite IRA-400) was used by Cao et al. (2002) to recover 1G-lactic acid from fermentation broth. The maximum adsorption capacity of lactic acid was observed at pH 2.0 (106 mg/g wet resin) and pH 5.0 (197.09 mg/g wet resin). However, during the column separation step, the total yield of lactic acid recovery at pH 2.0 (92.11%) was higher than pH 5.0 (86.21%). Consequently, the effect of pH on lactic acid adsorption is highly depend on the type the resins, whether it is strong, moderate, or weak base resins.

#### Effect of temperature on lactic acid adsorption

Since the adsorption rate depended on temperature, several studies had mainly investigated its effect on lactic acid recovery. Ataei and Vasheghani-Farahani (2008) stated that the temperature of 37 °C was the best condition for 2G-lactic acid adsorption by resin Amberlite IRA-400 with lactic acid production of 37.4 g/L. However, according to Srivastava et al. (1992), the fermentation productivity was reduced at a high temperature (> 39 °C) due to the low capacity of resin to adsorb lactate ions. The study reported that the best growth condition for lactic acid-producing organisms is at 37 °C, although lactic acid production was optimum at 44 to 45 °C. Therefore, the optimized temperature for lactic acid production is set at 39 °C due to the two temperature-dependent processes' combined effect; where the 1G-lactic acid yield and productivity was reported to be improved at the determined temperature. Whereas, it was notable that the amount of adsorbed 2G-lactic acid in IRA-400 decreased with the increase of temperature (Yuwono et al. 2017) and the result was supported by Gao et al. (2010) that observed a reduction in lactic acid adsorption (~ 20%) from the fermentation broth when the temperature was increased from 20 to 50 °C, using Amberlite IRA-67 as the adsorbent. Also, Nam et al. (2011) has study the adsorption equilibrium of lactic acid and succinic acid separation from fermentation broth in a quite similar temperature range of 30 to 50 °C and result showed that both acids' adsorption equilibrium was affected by temperature, where the increase in temperature had reduced (~ 20 to 26%) the lactic acid adsorption onto Amberchrom CG300C resin. Then, Uslu and Majumder (2017) studied the feasibility of using ion-exchange Amberlite XAD-7 for lactic acid adsorption from aqueous solution under various temperatures. The study observed a slight adverse effect of rising temperature (25, 35, and 45 °C) on lactic acid recovery, which reduced to about 6% to 7% only.

However, Moldes et al. (2003) and Zaini et al. (2019b) reported that the effect of temperature was not significant and had negligible influence on 2G-lactic acid adsorption capacities in anion-exchange resin. The stability of the resin across a wide range of temperature (30–80 °C) was observed by Pradhan et al. (2017), where the value for adsorption capacity of IRA-400 (24 mg/g) and recovery efficiency (~ 51%) remained constant. Next, Arcanjo et al. (2015) reported good performance of Amberlite IRA-67 and IRA-96, where both were not influenced by the increase in temperature during the lactic acid separation process. Thus, there are still arguments on the influence of temperature on the lactic acid recovery and separation process.

#### Effect of resin types on lactic acid adsorption

In terms of lactic acid separation, resin types also play an essential role, as different types of resin may function in different ways and favor different circumstances. Rincon et al. (1997) investigated various cation exchange resins (Amberlite IR-120, Amberlite 200, Amberlite 252, Lewatit S-100, and Dowex XVS) to separate 2G-lactic acid from highly concentrated lactic acid whey fermentation broth. The best resin chosen was Amberlite IR-120, a strong gel cation exchange resin as it possessed good physical strength, high capacity, and high exchange rate during lactic acid separation. Also, Amberlite IRA-96 studied by Arcanjo et al. (2015) showed a high adsorption capacity of 544 g/L with 99.2 wt% of the lactic acid recovery. Consequently, Bishai et al. (2015) used both exchange resins, Amberlite IRA-120 coupled with Amberlite IRA-96, to separate 2G-lactic acid from the fermentation broth with substrate from *Zizyphus oenophlia.* The maximum lactic acid-binding capacity was 210.46 mg lactic acid/g resin, whereas the purity of lactic acid increased to 99.17% with a final recovery yield of 98.9%.

Meanwhile, Amberlite IRA-67 has been reported numerous times to exhibit good performance in terms of high affinity, capacity, and vulnerability in 2G-lactic acid recovery. Moldes et al. (2003) compared different resin types, which included Amberlite IRA-900, IRA-400, IRA-96, and IRA-67. The resins physicochemical properties such as adsorption capacity, kinetics, and selectivity were evaluated and Amberlite IRA-67 (Cl^−^ form) was selected as the best resin for 2G-lactic acid recovery. The result was supported by Bernardo et al. (2016), who also investigated the production of 2G-lactic acid by *Lactobacillus rhamnosus* B103 from industrial dairy waste. Two resins, Amberlite IRA-67 and IRA-400, were compared for 2G-lactic acid adsorption and recovery, where the results showed that although both resins exhibited high adsorption capacity, Amberlite IRA-67 has better performance compared to Amberlite IRA-400 for 2G-lactic acid recovery (13.4%), with 100% protein and color removal as well as 99.10% of sugar removal. John et al. (2008) compared 2G-lactic acid recovery from fermented cassava bagasse using strong anion-exchange resin (Amberlite IRA-402) and weak anion-exchange resin (Amberlite IRA-67). The binding of lactic acid capacities (93%) of Amberlite IRA-67 (Cl- form) in fermentation broth has a more promising result and more efficient than Amberlite IRA-402. Apart from that, Luongo et al. (2019) studied the adsorption ability of anion-exchange resin (Amberlite IRA-900, IRA-400, IRA-96, and IRA-67) to recover lactic acid from solutions that mimic *Thermotoga neapolitana* fermentation broth. Even after 13 cycles of adsorption–desorption batch experiments, the tertiary amine-based resin IRA-67 showed the highest average lactic acid removal efficiency of 97% and it also exhibited the highest stability and selectivity among resin tested throughout the process. Bayazit et al. (2011a) has compared the lactic acid adsorption efficiency of Amberlite IRA-67 with activated carbon and IRA-67 resin was proven to be more effective for lactic acid adsorption in the fermentation broth compared to activated carbon, with maximum adsorption capacity for IRA-67 was 89.09% (220.69 mg/g absorbed lactic acid) which was much higher than the activated carbon (31.81%, equivalent to 157.64 mg/g absorbed lactic acid).

Other types of resin are also intensively investigated by many researchers. For example, Cui et al. (2016) evaluated different types of anion-exchange resins to remove 1G-lactic acid from a fed-batch culture of *Lactobacillus plantarum*. Among the tested resins, D319 (OH^−^ form) was selected to be used in the lactic acid removal system as it demonstrated good adsorption capacity for lactic acid. The result observed that the resin could remove lactate from the fermentation broth and slow down the osmotic pressure that increased throughout the fermentation. Generally, the study reported a potential high-density cultivation technique, in which the resin selection is a significant factor that permits better LAB growth; about 2.3-fold enhancement compared to fermentation without resin. The technique has been very substantial in producing many modern bioproducts, including lactic acid. Other than that, Yuwono et al. (2017) had purified 2G-lactic acid from cassava bagasse fermentation of *Streptococcus bovis* using ion-exchange resin of Amberlite IRA A 400, SA 10A, WA30, WK 10, and PK 228. The result showed that ion-exchange resin WA30 has the highest lactic acid adsorption amount in the lactic acid solution's batch adsorption, with lactic acid recovery yield of 60% and 80% purity rate. Recently, Zhang et al. (2018) has purified 1G-lactic acid from fermentation by *Bacillus coagulans* using weak base anion-exchange resin (D354, D380, D941, D396, D293, D301, D315, 335, D201, and 717). Among tested resins, resin 335 has exhibited the highest lactic acid adsorption capacity (402 mg/g) with high lactic acid recovery (~ 82%) even after ten times usage, and the final lactic acid production was about 0.96 g/L.

Thus, the lactic acid recovery can be carried out using either a strong base resin (e.g., Amberlite IRA-400 and IRA-120) or a weak base resin (e.g., Amberlite IRA-67 and IRA-96). Although numerous studies use weak base resin compared to strong base resin, several studies have combined these two resin types for better lactic acid separation and purification.

#### Effect of desorption solution on lactic acid recovery

The other factor that plays an essential role in lactic acid purification is the desorption reagent used to recover lactic acid from resin in the elution step. Several solvents and reagents were investigated for sufficient lactic acid detachment from resin. Quintero et al. (2012) used Amberlite IRA-400 and IR-120 to recover 2G-lactic acid from high concentration lactic acid (> 50 g L^−1^) fermentation broth of *Lactobacillus brevis.* The highest lactic acid productivity was 0.20 g/Lh with high lactic acid recovery (92.7% ± 1.9) was observed when 1 M NaOH was used as a desorbing agent compared to other eluents (0.1 M HCl, 10% methanol, 1 M H_2_SO_4_, and 1 M NaCl). However, Luongo et al. (2019) had successfully recovered lactic acid using Amberlite IRA-67 with a lower NaOH concentration (0.5 M) by three-bed volumes of desorbing solvent, resulted in 63% desorption efficiency and 30 g/L concentration of lactic acid. In addition, Pleissner et al. (2017) reported the usage of FPA 53 resin and 12.5 mmol L^−1^ H_2_SO_4_ were able to separate the 1G-lactic acid and salts-ions with more than 90% of lactic acid was successfully recovered, and the lactic acid production was achieved at 92.5 g/L. Meanwhile, Boonmee et al. (2016) reported the use of 1 M HCl at a low flow rate of only 0.1 BV/min, which resulted in 80% 1G-lactic acid recovery compared to resin-bound lactate. Recently, the study by Delgado et al. (2018) proposed the model with simulated moving bed (SMB) process for lactic acid separation using polyvinyl pyridine resin (Reillex 425) from aqueous solution (5% w/w lactic acid) and methanol as eluent for desorption process. The simulation's final result showed a higher lactic acid concentration (80% in water) and high recovery of 95%, as well as productivity of 22.7 kg m_solid_^−3^ h^−1^ with methanol consumption of only 0.062 m^3^ kg_lactic acid_^−1^.

#### In-site separation of lactic acid during fermentation

A vital need in the industry is to upgrade or improve lactic acid recovery technique from fermentation broth. Innovative approaches that can reduce the operational, labor, and maintenance costs are of interest. One of the potential approaches that can be taken is by incorporating ion-exchange resin inside the fermentation broth during the fermentation process. This simultaneous production and adsorption of lactic acid are known as in-site separation. Here, lactic acid produced by lactic acid bacteria will simultaneously be separated from the fermentation broth by adsorption to anion-exchange resin. No neutralizing agent is needed to control the pH of fermentation broth, as lactic acid produced is immediately bound to the resin.

Boonmee et al. (2016) studied in-site separation of 1G-lactic acid using anion-exchange resin Amberlite IRA-67. About 5.9-fold increase of lactic acid productivity was observed compared to basic batch fermentation without resin addition. The total recovery of lactate was recorded about 70% of the total lactate produced during fermentation. Meanwhile, Rampai et al. (2016) has studied the improvement of 1G-lactic acid production by simultaneous recovery during batch fermentation using Amberlite IRA-400. Approximate amount of IRA-400 was added to fermentation broth at pH 6.0 (70 ℃) and result showed that the lactic acid separation rate was around 1.8 g lactic acid/g wet resin. On the other hand, Othman et al. (2017b) and Cui et al. (2016) have developed a high-density culture strategy to improve *Pediococcus acidilactici* and *Lactobacillus plantarum* growth, respectively, by overcoming lactic acid inhibition using the in-site separation method. The strategy used a practical approach with minimal and simple process control equipment. In-site separation of IRA-67 Othman et al. (2017a, b) in a batch fermentation exhibited about 55.5 times increase of viable cell concentration, which were also higher than in fed-batch fermentation by 9.1 times. In the end, this study emphasized that the enhancement of viable cell concentration is proportional to the lactic acid production of probiotics. After that, Othman et al. (2018) further studied the enhancement of similar probiotic growth using extractive batch fermentation of ion-exchange resin to reduce by-product inhibition and increase lactic acid production. The study observed a good lactic acid adsorption capacity of 0.996 g lactic acid/g wet resin, whereas the in-site addition of 10 g/L Amberlite IRA-67 improved the growth of probiotic about 67 times compared to normal fermentation without the addition of resin. The growth was further enhanced by 1.4 times when integrated with a bioreactor–internal column system containing anion-exchange resin. The final 1G-lactic acid productivity reported from the study was about 0.59 g/L. Although recent studies conducted only focused on 1G-lactic acid separation and recovery from fermentation broth but, there are big opportunities for this methodology to be applied in 2G-lactic acid recovery as this technique renders several crucial advantages such as higher feedstock utilization, productivity improvement, as well as reduction of downstream load and recovery cost.

### Advantages of ion-exchange resin

Ion-exchange resins are essential polyelectrolytes, which can have two ions of opposite charge in a molecule. By having these ions, effective ionic compounds fractionation can be achieved, especially during the purification stage. For instance, Hughes et al. (2016) had developed a rapid, semi-automated method to fractionate dissolved organic carbon of freshwater using XAD 8 and XAD 4 resins in tandem. In contrast, conventional electrolytes only have one ion charge fixed to the polymeric or crystalline structure. The resin was reported to exhibit high stability and good exchange capacities, making it highly attractive to be used as an adsorbent (Seeber et al. 1998). Ion-exchange resin can dissociate and take part in the ion-exchange resin processes without altering its physical state. Therefore, it has a high potential to be utilized to design a heterogeneous system of ion exchange processes with this ionic property of resins (Kumar and Jain 2013). Next, ion-exchange resin can convert lactate salt to lactic acid for lactic acid recovery. The adsorption of lactic acid is also highly selective, and the time required to recover lactic acid is shorter compared to the other purification methods. Ion-exchange resin also requires lower running costs, as only little energy is required with no phase transition throughout the separation process (Park et al. 2014). The cheaper regenerant chemicals needed for the operation and resin beds can be well maintained, causing it to last for many years before replacement is necessary. This method can significantly reduce the cost of neutralizing chemicals, exhibiting industrial applications' potential (Zhang et al. 2018). Furthermore, ion-exchange resins are physically and chemically stable and insoluble in acid, alkali, or organic solvents. Besides, most anionic resins can be used directly in the fermenter, as they are non-toxic to microorganisms (Nielsen et al. 2010). The purification process using ion-exchange resins is simple, more manageable, cheaper, and does not require expensive equipment (Ghaffar et al. 2014; Quintero et al. 2012).

### Limitation of adsorption technique by ion-exchange resin

However, some limitations are associated with lactic acid purification by ion-exchange. According to Aljundi et al. (2005), ion exchange generates a large volume of waste liquor and massive acid usage and base in the elution process. This notion was supported by Boonmee et al. (2016), who stated that the elution step produced a large amount of water and caused a liquid waste stream. Moreover, the resin exchange capacity will be weakened with extended time (Aljundi et al. 2005). In addition, the existence of nutrients and ions other than lactate, e.g., acetic acid, sodium acetate, and glucose, may slightly decrease the capacity of resin in simultaneous saccharification and fermentation media (SSF) (Moldes et al. 2003; Boonmee et al. 2016).

## Lactic acid recovery using other techniques

Apart from ion-exchange adsorption, other techniques for lactic acid recovery from fermentation broth have been proposed previously, including conventional chemical neutralization, liquid–liquid extraction (Matsumoto et al. 2016), membrane separation (Alexandri et al. 2018), and distillation (Rao et al. 2014). The overall summary for all lactic acid separation and recovery process is tabulated in Table 2. Each of the processes has its advantages and disadvantages to be applied and operated efficiently, particularly for high-purity lactic acid production.

**Table 2:** Summary of lactic acid separation and recovery process

Separation/recovery process	Advantages	Disadvantages	References
Chemical neutralization/precipitation	Relatively simple operation process System is applicable in industrial High product yield	Production of gypsum waste which causes an environmental problem High solvent consumption (e.g., sulphuric acid or calcium hydroxide) Low product purity	(Daful et al. 2016; Komesu et al. 2017a; Wasewar et al. 2003; Yankov et al. 2005)
Ion exchange adsorption	No waste generation production Simple and not require expensive equipment operation High stability and selectivity of operation Shorter time for product recovery Low running costs and energy consumption Non-toxic properties to microorganisms High potential to integrate into a heterogeneous system Resin can be regenerated to be reuse	Large waste liquor due to massive usage of eluent Operation not suitable for high temperature and long-term production Problem with co-extraction of other compounds	(Pradhan et al. 2017; Seeber et al. 1998; Kumar and Jain 2013; Aljundi et al. 2005; Boonmee et al. 2016; Zhang et al. 2018; Nielsen et al. 2010; Quintero et al. 2012)
Liquid–liquid extraction	Gypsum waste is not produced Low risk of thermal decomposition High selectivity Low end-product inhibition	Low product recovery due to stepwise evaporation or re-extraction step High solvent cost consumption Low product purity May cause intoxication in the system Risk of co-adsorption	(Komesu et al. 2017a; López-Garzón and Straathof 2014; Wasewar et al. 2003; Henczka and Djas 2016)
Membrane extraction	High adaptability and selectivity of the recovery process High product purity High flexibility as it is easy to scale up Effective elimination of impurities	Expensive cost of membranes Problems with polarization Membrane fouling problem Retention of lactic acid	(Wojtyniak et al. 2016; Komesu et al. 2017a; Kumar et al. 2019)
Distillation	No use of solvents More efficiency to produce high product purity	Process is complex Difficult to scale up Process requires specific temperature and pressure conditions with high operational cost Mismatch problem with optimum process conditions Problems with volatility constraints	(Komesu et al. 2017b; Aqar et al. 2016; Wojtyniak et al. 2016)

### Chemical neutralization

Chemical neutralization by precipitation is the earliest technique used in industrial plants to neutralize broth during LAB fermentation (Zhang et al. 2019a, b). This method employed chemical base such as calcium hydroxide (Ca[OH]_2_) and calcium carbonate (CaCO_3_) to overcome the inhibitory effect of undissociated lactic acid formed in the broth by converting it to acid salt (Patel et al. 2008). These two chemicals are the most used base in the industry, facilitating the downstream process operation. Calcium salt precipitation will be formed at the end of the process (Wasewar et al. 2004) and filtered to separate it from the broth. Next, acidification of broth with sulphuric acid (H_2_SO_4_) will produce calcium sulfate (gypsum), as the precipitate along with lactic acid, which is then recovered in the filtrate (Ameen and Caruso 2017). The diluted lactic acid (technical grade ~ 22%–44%) is then purified sequentially by activated carbon, evaporation, and crystallization process (Alves De Oliveira et al. 2020; Komesu et al. 2017b).

However, the use of calcium hydroxide as a neutralizing agent causes several drawbacks, especially the formation of a large amount of gypsum, an environmental pollutant discarded as solid waste. Datta et al. (1995) reported that almost one ton of gypsum is produced for every ton of lactic acid produced by a conventional method, and it needs to be disposed of to the environment. While gypsum is used in the manufacture of the board, cement, pharmaceuticals, and also as fertilizers, the production of lactic acid that produces less or no gypsum is more in demand, as it can prevent costly treatment of gypsum before disposal, which accounted for more than 50% of the production cost (Seong et al. 2016; Boonmee et al. 2016; Wasewar et al. 2003). Other than that, the process also consumes a high amount of sulphuric acid and calcium hydroxide that contributed to environmental problems (Othman et al. 2017a). Apart from that, Gao et al. (2009) found that calcium lactate was formed in a colloidal state, which caused flocculation of fermentation broth when the calcium lactate level is high and eventually cause incomplete fermentation. Consequently, low lactic acid purity was produced at the end of the precipitation process (Wasewar 2005; Komesu et al. 2017b).

Nevertheless, several improvements to the precipitation method have been developed. For example, Kwak et al. (2012) studied the effect of methanol addition during ammonium lactate acidification, which resulted in the reduction of ammonium sulfate solubility in broth, and its separation could be facilitated using simple filtration. Moreover, the methyl lactate can easily be distilled to obtain lactic acid at room temperature, providing a yield of more than 80%. Meanwhile, the by-product of ammonium sulfate can be utilized for sulphuric acid and ammonia production. Recently, the separation of lactic acid in terms of magnesium precipitation technology has emerged as a potential lactic acid separation method (Zhang et al. 2019a, b). Magnesium hydroxide (Mg[OH]_2_) has been used as a neutralizing agent and generates magnesium lactate, which further reacts with trimethylamine to yield a complex of trimethylamine–LA and crystals that can be recovered by filtration for further use. The complex is then thermally decomposed to produce lactic acid, and trimethylamine can be recycled into the fermentation process (Daful et al. 2016). The processes are relatively cost-effective, as the cost for precipitation management is reduced, and the by-products can be reused.

### Liquid–liquid extraction

The liquid–liquid extraction of lactic acid is principally based on a separation of the compound and their relative solubility in two different types of immiscible liquid. Conventional liquid–liquid extraction uses diethyl ether, decanol, octanol, chloroform, hexane, and tertiary amines as extracting solvent. Recently, alternative approaches to conventional solvent extraction, such as reactive extraction, ionic solvent extraction, and aqueous two-phase system (ATPS), have been studied extensively. These methods usually have short cycles and faster mass transfer between the liquid than solid phases (Li et al. 2016).

In the reactive extraction method, the reactions occur between extractants and solute molecules extracted and eventually produce a specific chemical compound or association complex. Then, the chemical is further solubilized in the organic phase (Antony and Wasewar 2019). The process converts the product into other compounds such as ester to allow for a more straightforward extraction step. Chemicals such as hydrocarbon solvents, phosphorus solvents, and aliphatic amines were among the extractants used for lactic acid separation from fermentation broth (Bayazit et al. 2011b; Yankov et al. 2005). Hu et al. (2017) used ethyl acetate (ultrasonic-mediated) for lactic acid extraction from three different fermented broth kinds, i.e., commercial glucose, mixed food waste hydrolysate, and bakery waste hydrolysate. The result showed high lactic acid extraction yields (92%–93%) with a total recovery of 82%–84%. The impurities were removed, and high-purity lactic acid (~ 98%) was obtained. Natural solvents have recently been studied as extractants for organic acid recovery (De et al. 2018). Besides, reactive extraction can also be integrated as in-site process, which helps produce higher product yield. In the in-site reactive extraction process, a solution is used in organic acid production to reduce product inhibition and enhance productivity (Zhang and Yang 2015); which can reduce the toxic effect of extractants by shifting the undesirable reaction equilibrium, reducing product degradation, and decreasing the downstream process routes (Wee et al. 2006; Ataei and Vasheghani-Farahani 2008).

Meanwhile, the ionic liquid extraction is the separation technique using organic salts (ionic liquid) such as imidazolium (Lateef et al. 2012), quaternary phosphate (Oliveira et al. 2012) or quaternary ammonium salts (Kulkarni et al. 2007), which are chemically stable, non-volatile, non-flammable, lower viscosity, and higher density than many organic solvents (Li et al. 2016). The examples of ionic liquid that function as good extractant for lactic acid are imidazolium-based ionic liquid (Lateef et al. 2012) and phosphonium-based ionic liquid (Oliveira et al. 2012). The ATPS has unique features as the solvents become aqueous solutions either of two polymers (e.g., polyethylene glycol [PEG] and dextran) or a polymer and a salt (e.g., sulfate, phosphate, or citrate) (Goja et al. 2013; Iqbal et al. 2016). Aydoğan et al. (2011) reported the use of alcohol/salt ATPS for lactic acid extraction. The purification process was optimized by response surface methodology to determine ethanol/dipotassium hydrogen phosphate usage for lactic acid recovery. Up to 80% lactic acid extraction yield was recovered in this study (Aydoğan et al. 2011).

However, the disadvantages of using this method for lactic acid separation are mainly due to the hydrophilic nature of organic solvents (Gao et al. 2010). Aydoğan et al. (2011) reported that up to 85% of extracted lactic acid was lost during lactic acid recovery during the isolation step, which required stepwise evaporation or re-extraction using hydrochloric acid. Moreover, a high amount of solvents were typically used, and the in-site solvents are toxic, as it can rupture the cell membrane, causing leaked metabolites, as well as disturbances of essential membrane functions, membrane-bound enzymes denaturation, transport mechanisms breakdown, and even solvolysis of cells at high concentrations (Othman et al. 2017a; Aljundi et al. 2005; Marinova and Yankov 2009). Besides, various diluents added in the process may cause pollution to the environment. Apart from that, the ionic liquid and polymers used in prospective lactic acid extraction ATPS are not economically feasible due to high costs (Aydoğan et al. 2011; Li et al. 2016).

### Membrane extraction

The advantages of the membrane extraction method are its adaptability and selectivity in the recovery process. A membrane is a thin barrier (natural or artificial) that acts for physical separation or enrichment purposes by controlling the selective mass transport of solutes or solvents across the barrier. This method could produce high-purity target products because of its high selectivity and flexibility. The types of membrane filtrations used for separation are microfiltration, nanofiltration, ultrafiltration, reverse osmosis, pervaporation, and electrodialysis (Mao et al. 2014; Cheng et al. 2012; Vane 2005). The membrane can be incorporated into conventional fermenters, permitting simultaneous production and purification (Pal et al. 2009).

The first model of lactic acid transportation through nanofiltration and reverse osmosis (RO) membranes was developed by Timmer et al. (1993). The model was designed based on the extended Nernst–Planck equation, whereby lactic acid was described to be transported through nanofiltration and RO membranes in a spiral wound module where the form of lactic acid is based on the pH of the buffer. A recent study by Alexandri et al. (2018) on the separation of lactic acid from fermented broth and other fermentation components by both microfiltration and nanofiltration found that the microfiltration efficiently separate lactic acid from cell biomass and other high molecular weight particles, such as sugars, proteins, and metal ions with minor losses (~ 16%–21%). It led to a 13% improvement in lactic acid purity. Nanofiltration was also applied by Oonkhanond et al. (2017) to separate lactic acid from sugarcane bagasse cellulosic hydrolysate. The study reported that the low flux nanofiltration membrane gave the highest efficiency compared to the high flux membrane. Approximately 93.3% of lactic acid was separated with 82.5% membrane selectivity. Apart from that, Neu et al. (2016) incorporated microfiltration and nanofiltration as the pre-treatment for lactic acid separation from coffee mucilage fermentation broth. After the first filtration step, about 12.6% of lactic acid was lost in the retentate stream. However, the lactic acid recovery was increased by approximately 20% in the retentate stream after nanofiltration.

Meanwhile, electrodialysis is applied to concentrate ionic compounds and to remove salts from solutions. Bernardo et al. (2016) stated that the electrodialysis method provided fast treatment, concentrated lactic acid with the effective removal of non-ionic molecules, and an efficient working process. Whereas, in-site removal of lactic acid used electrodialysis fermentation coupled with ion exchange membrane to remove ions from the aqueous solution under the driving force of electrical fields (Habova et al. 2004; Wasewar 2005). The good bipolar membrane feature used in a two-stage electrodialysis developed by Habova et al. (2001) was applied for in-site lactic acid from *Lactobacillus plantarum* L10 fermentation. Methodologically, the first stage involved concentration by desalting electrodialysis using ion-exchange membranes, which produced the highest lactate concentration of 111 g/L (an increase of more than 2.5-times from the initial concentration), followed by electro conversion of sodium lactate to lactic acid in the second stage by water-splitting electrodialysis with the bipolar membrane, giving the final concentration of lactic acid of 157 g/L. However, the whole separation process recorded high energy consumption of 1.5 kWh per 1 kg and was regarded as a drawback (Habova et al. 2004). Nonetheless, later studies managed to complete the process with lower energy consumption, e.g., Chen et al. (2016) only used 0.014 kW to separate lactic acid from 1 kg of whey, with 90% acid demineralization. The method's cost concerns also included operational factors such as water transfer and concentration polarization (Wasewar et al. 2004; Boontawan et al. 2011; Wang et al. 2013).

Furthermore, Lech and Trusek (2018) reported that the most significant problem of the method was the presence of other acids in the receiving chamber, in which the quality of the broth highly influences their concentrations. The membrane separation efficiency might be reduced as organic acid concentration increases. The membrane fouling also occurred during electrodialysis as other substances, including acids, can block the membrane surface, which could be caused by proteins in the fermentation broth and the increase in cell numbers. The integration of the electrodialysis system into fermentation resulted in the attachment of cells to the membrane, and microfiltration could be applied before electrodialysis to separate the cells from broth (Boonmee et al. 2007; Boontawan et al. 2011). However, membrane fouling has a minimum effect on the process (Lech and Trusek 2018). Apart from the expensive cost of the membrane, the membrane pollution that resulted in the production of by-product salt formation during the ion-exchange process is another disadvantage of the method (Wasewar 2005; Boonkong et al. 2009).

### Distillation

The distillation method is a practical separation method of a mixture according to the variances in substance volatility, and it can be applied before or after the separation of lactic acid. It is a powerful method in the refining step, although various other methods are capable of separating the substance. Like lactic acid, most organic acids have higher boiling points compared to water due to the strong adsorb-electron effect by the carbonyl group in their structure. Thus, conventional distillation carried out at normal temperature and pressure is inadequate for lactic acid recovery from the fermentation broth (Li et al. 2016). The problem can be overcome by reactive and extractive distillation, as it involved the conversion of crude lactic acid to esters, followed by hydrolysis into lactic acid in the distillation unit. It produced highly pure lactic acid with more efficiency.

Earlier, Schopmeyer and Arnold (1944) patented the method for a continuous process where crude lactic acid reacts with methanol in the presence of sulfuric acid as the homogenous catalyst. However, the method had problems with corrosion and separation, as well as side reactions of the homogenous catalyst, making it difficult for industrial applications. Later, the problem was solved by developing a batch reactive distillation system using cation exchange resins, consisting of esterification and hydrolysis reactor along with a fractionating column (Choi and Hong 1999). Rao et al. (2014) applied reactive distillation to recover lactic acid, and the yield from the process was estimated to be 95% when all evaporated water was condensed and removed from the distillation system. Reactive distillation benefits include low esterification equipment cost, better reactants conversion and selectivity of products, low catalyst amount requirement, and heat integration for an exothermic reaction (Komesu et al. 2017b; Aqar et al. 2016).

On the contrary, several difficulties in performing reactive distillation were reported (Wojtyniak et al. 2016). There are volatility constraints, as high concentrations of reactants and low concentrations of products must be maintained in the reaction zone. Apart from that, high investment at the initial stage of process start-up is one of the most common problems with molecular distillation (Xu et al. 2018; Breisig et al. 2017). The process's efficiency is reduced at a high concentration of the products, especially at conditions approaching azeotropic point (Huang et al. 2008). Besides, the extended retention time of the process requires large tray holdups, which is economically unattractive compared to the usage of the reactor–separator arrangement. The liquid distribution problem in the packed reactive distillation causes difficulty in planning the method for substantial flow rates. Meanwhile, the optimum process conditions of temperature and pressure may mismatch for reaction and vice versa in some processes of reactive distillation (Komesu et al. 2017b).

## Conclusion and perspectives

Fermentation of feedstock waste is a promising method for lactic acid production. However, lactic acid separation and recovery during the downstream process are complicated and expensive. Ion-exchange resin is one of the most prevalent and straightforward extractive fermentation strategies used to avoid the shortcomings of lactic acid production. Resin is effectively applied as a lactic acid adsorbent, as it exhibits high capacity and good selectivity for the lactic acid adsorption process. Thus, high lactic acid production with good purity can be achieved in the final stage of adsorption, as reported in many previous studies. The recent use of resin by in-site separation was proven to significantly increase the lactic acid production by providing a suitable pH environment for the growth of LAB and at the same time reducing the inhibitory effect of lactic acid accumulation in the fermentation broth. It is expected to be an effective operative process with low energy and cost consumption compared to conventional approaches. Meanwhile, other extractive fermentation strategies such as distillation, membrane extraction, and liquid–liquid extraction are also useful for lactic acid recovery from fermentation broth. Nevertheless, all processes have their limitations, and it is still a challenge to develop a scale-up methodology that could efficiently produce and recover high yield pure lactic acid at the minimum labor and operation cost. Therefore, the choice of recovery and purification strategies is dependent on the types of raw material used, operation cost, and maintenance.

## Data Availability

All data generated or analyzed during this study are included in this published article.

## Abbreviations

GRAS: Generally recognized as safe
FDA: Food and Drug Administration
CAGR: Compound annual growth rate
USD: United States dollar
GMO: Genetically modified organism
PLA: Poly-lactic acid
LAB: Lactic acid bacteria
PET: Polyethylene terephthalate
PEG: Polyethylene glycol
HMF: Hydroxymethyl furfural
BV: Bed volume
SMB: Simulated moving bed
1G: First generation
2G: Second generation
ATPS: Aqueous two-phase system
RO: Reverse osmosis
OD: Optical density
CFU: Colony-forming unit

## References

[CR1] Abdel-Rahman MA, Sonomoto K (2016). Opportunities to overcome the current limitations and challenges for efficient microbial production of optically pure lactic acid. J Biotechnol.

[CR2] Abdel-Rahman MA, Tashiro Y, Sonomoto K (2013). Recent advances in lactic acid production by microbial fermentation processes. Biotechnol Adv.

[CR3] Abdulaziz M, Musayev S (2017). Multicomponent biosorption of heavy metals from aqueous solutions: A review. Pol J Environ Stud.

[CR4] Alexandri M, Schneider R, Venus J (2018). Membrane technologies for lactic acid separation from fermentation broths derived from renewable resources. Membranes.

[CR5] Aljundi IH, Belovich JM, Talu O (2005). Adsorption of lactic acid from fermentation broth and aqueous solutions on Zeolite molecular sieves. Chem Eng Sci.

[CR6] Alves De Oliveira R, Alexandri M, Komesu A, Venus J, Vaz Rossell CE, Maciel Filho R (2020). Current advances in separation and purification of second-generation lactic acid. Sep Purif Rev.

[CR7] Alvira P, Tomás-Pejó E, Ballesteros M, Negro M (2010). Pretreatment technologies for an efficient bioethanol production process based on enzymatic hydrolysis: a review. Biores Technol.

[CR8] Ameen SM, Caruso G, Caruso G, Ameen SM (2017). Chemistry of Lactic Acid. Lactic Acid in the Food Industry.

[CR9] Antony FM, Wasewar K (2019) Reactive extraction: a promising approach to separate protocatechuic acid. Environmental Science Pollution Research:1–13 10.1007/s11356-019-06094-x 31388958

[CR10] Aqar DY, Rahmanian N, Mujtaba IM (2016). Integrated batch reactive distillation column configurations for optimal synthesis of methyl lactate. Chem Eng Process Intensif.

[CR11] Arcanjo M, Fernandes F, Silva I (2015). Separation of lactic acid produced by hydrothermal conversion of glycerol using ion-exchange chromatography. Adsorpt Sci Technol.

[CR12] Ataei SA, Vasheghani-Farahani E (2008). In situ separation of lactic acid from fermentation broth using ion exchange resins. J Ind Microbiol Biotechnol.

[CR13] Aydoğan Ö, Bayraktar E, Mehmetoğlu Ü (2011). Aqueous two-phase extraction of lactic acid: Optimization by response surface methodology. Sep Sci Technol.

[CR14] Bai Z, Gao Z, He B, Wu B (2015). Effect of lignocellulose-derived inhibitors on the growth and  d-lactic acid production of* Sporolactobacillus inulinus* YBS1-5. Bioprocess Biosystems Eng.

[CR15] Batubara F, Selviani C, Turmuzi M, Majlan EH (2019). Effect of Cu-purolite A400 resin on adsorption of nitrate and nitrite in wastewater treatment. Malaysian J Anal Sci.

[CR16] Bernardo MP, Coelho LF, Sass DC, Contiero J (2016). l-(+)-Lactic acid production by *Lactobacillus rhamnosus* B103 from dairy industry waste. Braz J Microbiol.

[CR17] Biddy MJ, Scarlata C, Kinchin C (2016). Chemical from bBiomass: A market assessment of bioproducts with near-term potential (Technical Report NREL/TP-5100-65509).

[CR18] Bio-Resource (2011) Ion Exchange Chromatography Principle. http://technologyinscience.blogspot.com/2011/09/ion-exchange-chromatography-principle.html#.X14JPWgzbIU. Accessed January 13, 2020 January 13, 2020

[CR19] Bishai M, De S, Adhikari B, Banerjee R (2015). A platform technology of recovery of lactic acid from a fermentation broth of novel substrate* Zizyphus oenophlia*. 3 Biotech.

[CR20] Boonkong W, Sangvanich P, Petsom A, Thongchul N (2009). Comparison of an ion exchanger and an in-house electrodialysis unit for recovery of l-lactic acid from fungal fermentation broth. Chem Eng Technol.

[CR21] Boonmee M, Leksawasdi N, Bridge W, Rogers PL (2007). Electrodialysis for lactate removal in the production of the dairy starter culture* Lactococcus lactis* NZ133. Int J Food Sci Technol.

[CR22] Boonmee M, Cotano O, Amnuaypanich S, Grisadanurak N (2016). Improved lactic acid production by in situ removal of lactic acid during fermentation and a proposed scheme for its recovery. Arab J Sci E ng.

[CR23] Boontawan P, Kanchanathawee S, Boontawan A (2011). Extractive fermentation of l-(+)-lactic acid by* Pediococcus pentosaceus* using electrodeionization (EDI) technique. Biochem Eng J.

[CR24] Breisig H, Schmidt M, Wolff H, Jupke A, Wessling M (2017). Droplet-based liquid–liquid extraction inside a porous capillary. Chem Eng J.

[CR25] Broadbent JR, Larsen RL, Deibel V, Steele JL (2010). Physiological and transcriptional response of* Lactobacillus casei* ATCC 334 to acid stress. J Bacteriol.

[CR26] Buyondo JP, Liu S (2011). Lactic acid production by* Lactobacillus pentosus* from wood extract hydrolysates. J Sci Technol Forest Products Process.

[CR27] Cao X, Yun HS, Koo YM (2002). Recovery of* l*-(+)-lactic acid by anion exchange resin Amberlite IRA-400. Biochem Eng J.

[CR28] Chahal S (1990). Lactic acid.

[CR29] Chen GQ, Eschbach FI, Weeks M, Gras SL, Kentish SE (2016). Removal of lactic acid from acid whey using electrodialysis. Sep Purif Technol.

[CR30] Chen K, Hao S, Lyu H, Luo G, Zhang S, Chen J (2017). Ion exchange separation for recovery of monosaccharides, organic acids and phenolic compounds from hydrolysates of lignocellulosic biomass. Sep Purif Technol.

[CR31] Chen K, Luo G, Lei Z, Zhang Z, Zhang S, Chen J (2018). Chromatographic separation of glucose, xylose and arabinose from lignocellulosic hydrolysates using cation exchange resin. Sep Purif Technol.

[CR32] Cheng KK, Zhao XB, Zeng J, Wu RC, Xu YZ, Liu DH, Zhang JA (2012). Downstream processing of biotechnological produced succinic acid. Appl Microbiol Biotechnol.

[CR33] Choi JI, Hong WH (1999). Recovery of lactic acid by batch distillation with chemical reactions using ion exchange resin. J Chem Eng Jpn.

[CR34] Chowdhury R, Ghosh S, Debnath B, Manna D (2018) Indian Agro-Wastes for 2G Biorefineries: Strategic Decision on Conversion Processes. In: Sustainable Energy Technology and Policies. Springer, pp 353–373

[CR35] Couper JR, Penney WR, Fair JR, Couper JR, Penney WR, Fair JR (2012). Adsorption and ion exchange. Chemical Process Equipment - Selection and Design.

[CR36] Cubas-Cano E, González-Fernández C, Ballesteros M, Tomás-Pejó E (2018). Biotechnological advances in lactic acid production by lactic acid bacteria: lignocellulose as novel substrate. Biofuels Bioproduct Biorefining.

[CR37] Cui S, Zhao J, Zhang H, Chen W (2016). High-density culture of* Lactobacillus plantarum* coupled with a lactic acid removal system with anion-exchange resins. Biochem Eng J.

[CR38] Daful AG, Haigh K, Vaskan P, Görgens JF (2016). Environmental impact assessment of lignocellulosic lactic acid production: Integrated with existing sugar mills. Food Bioproducts Process.

[CR39] Datta R, Tsai SP, Bonsignore P, Moon SH, Frank JR (1995). Technological and economic potential of poly (lactic acid) and lactic acid derivatives. FEMS Microbiol Rev.

[CR40] De BS, Wasewar KL, Dhongde V (2018). Extractive separation of protocatechuic acid using natural non-toxic solvents and conventional solvents. Chemical Data Collections.

[CR41] de Jong E, Higson A, Walsh P, Wellisch M (2012) Bio-based chemicals value added products from biorefineries. IEA Bioenergy, Task42 Biorefinery:34

[CR42] Delgado JA, Águeda VI, Uguina MÁ, García Á, Matarredona J, Moral R (2018). Modeling of the separation of lactic acid from an aqueous mixture by adsorption on polyvinylpyridine resin and desorption with methanol. Sep Purif Technol.

[CR43] Di Cagno R, De Angelis M, Limitone A, Fox PF, Gobbetti M (2006). Response of* Lactobacillus helveticus* PR4 to heat stress during propagation in cheese whey with a gradient of decreasing temperatures. Appl Environ Microbiol.

[CR44] Dumbrepatil A, Adsul M, Chaudhari S, Khire J, Gokhale D (2008). Utilization of molasses sugar for lactic acid production by* Lactobacillus delbrueckii* subsp delbrueckii mutant Uc-3 in batch fermentation. Appl Environ Microbiol.

[CR45] Evangelista RL, Nikolov ZL (1996) Recovery and purification of lactic acid from fermentation broth by adsorption. Seventeenth Symposium on Biotechnology for Fuels and Chemicals:471–480

[CR46] Evangelista RL, Mangold AJ, Nikolov ZL Recovery of lactic acid by sorption. In: Applied biochemistry and biotechnology, 1994. vol 1. pp 131–144

[CR47] Even S, Lindley ND, Loubière P, Cocaign-Bousquet M (2002). Dynamic response of catabolic pathways to autoacidification in* Lactococcus lactis*: transcript profiling and stability in relation to metabolic and energetic constraints. Mol Microbiol.

[CR48] Fong E, Khan M, Aida WW, Maskat MY (2017). Effect of ion exchange resin weight and extract flow rate on the properties of starfruit (*Averrhoa carambola* L.) extract. Int Food Res J.

[CR49] Food and Drug Administration (2015) Lactic acid. Code of Federal Regulations Title 21. Food and Drug Administration (FDA), United States

[CR50] Gao MT, Hirata M, Toorisaka E, Hano T (2009). Development of a fermentation process for production of calcium-L-lactate. Chem Eng Process.

[CR51] Gao Q, Liu F, Zhang T, Zhang J, Jia S, Yu C, Jiang K, Gao N (2010). The role of lactic acid adsorption by ion exchange chromatography. PLoS ONE.

[CR52] Garba ZN, Zhou W, Lawan I, Xiao W, Zhang M, Wang L, Chen L, Yuan Z (2019). An overview of chlorophenols as contaminants and their removal from wastewater by adsorption: A review. J Environ Manage.

[CR53] Garrett BG, Srinivas K, Ahring BK (2015). Performance and stability of Amberlite^TM^ IRA-67 ion exchange resin for product extraction and pH control during homolactic fermentation of corn stover sugars. Biochem Eng J.

[CR54] Ghaffar T, Irshad M, Anwar Z, Aqil T, Zulifqar Z, Tariq A, Kamran M, Ehsan N, Mehmood S (2014). Recent trends in lactic acid biotechnology: a brief review on production to purification. J Radiation Res Appl Sci.

[CR55] Goja AM, Yang H, Cui M, Li C (2013). Aqueous two-phase extraction advances for bioseparation. J Bioprocess Biotechnol.

[CR56] González MI, Álvarez S, Riera FA, Álvarez R (2006). Purification of lactic acid from fermentation broths by ion-exchange resins. Ind Eng Chem Res.

[CR57] Grand Review Research (2019). Lactic Acid Market Size, Share & Trend Analysis Report By Raw Material (Corn, Sugarcane), By Application (Industrial, Food & Beverages, Polylactic Acid), By Region, And Segment Forecasts, 2019–2025.

[CR58] Gullón B, Alonso JL, Parajó JC (2010). Ion-exchange processing of fermentation media containing lactic acid and oligomeric saccharides. Ind Eng Chem Res.

[CR59] Habova V, Melzoch K, Rychtera M, Pribyl L, Mejta V (2001). Application of electrodialysis for lactic acid recovery. Czech J Food Sci.

[CR60] Habova V, Melzoch K, Rychtera M (2004) Modern method of lactic acid recovery from fermentation broth. Czech Journal of Food Sciences-UZPI (Czech Republic)

[CR61] Haslaniza H, Yaacob WW, Zubairi SI, Maskat MY (2015). Potential of Amberlite IRA 67 resin for deacidification of organic acids in noni juice. Der Pharma Chemica.

[CR62] Henczka M, Djas M (2016). Reactive extraction of acetic acid and propionic acid using supercritical carbon dioxide. J Supercritical Fluids.

[CR63] Hörhammer HS, Treasure TH, Gonzalez RW, van Heiningen AR (2014). Larch biorefinery: technical and economic evaluation. Ind Eng Chem Res.

[CR64] Hu Y, Kwan TH, Daoud WA, Lin CSK (2017). Continuous ultrasonic-mediated solvent extraction of lactic acid from fermentation broths. J Clean Prod.

[CR65] Huang HJ, Ramaswamy S, Tschirner UW, Ramarao B (2008). A review of separation technologies in current and future biorefineries. Sep Purif Technol.

[CR66] Hughes DD, Holliman PJ, Jones T, Freeman AJBC (2016). Rapid, semi-automated fractionation of freshwater dissolved organic carbon using DAX 8 (XAD 8) and XAD 4 resins in Tandem. Nat Sci.

[CR67] Idler C, Venus J, Kamm B, Kamm B (2015). Microorganisms for the production of lactic acid and organic lactates. Microorganisms in Biorefineries.

[CR68] Iqbal M, Tao Y, Xie S, Zhu Y, Chen D, Wang X, Huang L, Peng D, Sattar A, Shabbir MAB (2016). Aqueous two-phase system (ATPS): an overview and advances in its applications. Biological Procedures Online.

[CR69] Järvinen M, Myllykoski L, Keiski R, Sohlo J (2000). Separation of lactic acid from fermented broth by reactive extraction. Bioseparation.

[CR70] Jianlong W, Ping L, Ding Z (1994). Extractive fermentation of lactic acid by immobilized* Lactobacillus casei* using ion—exchange resin. Biotechnol Tech.

[CR71] Jianlong W, Xianghua W, Ding Z (2000). Production of citric acid from molasses integrated with in-situ product separation by ion-exchange resin adsorption. Biores Technol.

[CR72] John RP, Nampoothiri KM, Pandey A (2008). L (+)-Lactic acid recovery from cassava bagasse based fermented medium using anion exchange resins. Braz Arch Biol Technol.

[CR73] Jørgensen SE, Jørgensen SE, Gromiec MJ (1989). Adsorption and ion exchange. Mathematical Submodels in Water Quality Systems.

[CR74] Juodeikiene G, Vidmantiene D, Basinskiene L, Cernauskas D, Bartkiene E, Cizeikiene D (2015). Green metrics for sustainability of biobased lactic acid from starchy biomass vs chemical synthesis. Catal Today.

[CR75] Kammerer J, Carle R, Kammerer DR (2010). Adsorption and ion exchange: basic principles and their application in food processing. J Agric Food Chem.

[CR76] Karekar SC, Srinivas K, Ahring BK (2020). Continuous in-situ extraction of acetic acid produced by *Acetobacterium woodii* during fermentation of hydrogen and carbon dioxide using Amberlite FPA53 ion exchange resins. Bioresour Technol Rep.

[CR77] Khalafu SHS, Aida WMW, Lim SJ, Maskat MY (2017). Effects of deodorisation methods on volatile compounds, chemical properties and antioxidant activities of fucoidan isolated from brown seaweed (Sargassum sp.). Algal Res.

[CR78] Kim YH, Moon SH (2001). Lactic acid recovery from fermentation broth using one-stage electrodialysis. J Chem Technol Biotechnol Int Res Process Environ Clean Technol.

[CR79] Komesu A, Maciel MRW, Maciel Filho R (2017). Separation and purification technologies for lactic acid–A brief review. BioResources.

[CR80] Komesu A, Wolf Maciel MR, Rocha de Oliveira JA, da Silva Martins LH, Maciel Filho R (2017). Purification of lactic acid produced by fermentation: focus on non-traditional distillation processes. Sep Purif Rev.

[CR81] Koźlecki T, Sokołowski A, Wilk K (1997). Surface activity and micelle formation of anionic azobenzene-linked surfactants. Langmuir.

[CR82] Kulkarni PS, Branco LC, Crespo JG, Nunes MC, Raymundo A, Afonso CA (2007). Comparison of physicochemical properties of new ionic liquids based on imidazolium, quaternary ammonium, and guanidinium cations. Chem Eur J.

[CR83] Kulkarni SS, Juvekar VA, Mukhopadhyay S (2018). Intensification of emulsion liquid membrane extraction of uranium (VI) by replacing nitric acid with sodium nitrate solution. Chem Eng Process Intensif.

[CR84] Kulprathipanja S, Oroskar AR (1991) Separation of lactic acid from fermentation broth with an anionic polymeric absorbent. US Patent 4,323,702, 26 November 1991

[CR85] Kumar S, Jain S (2013) History, introduction, and kinetics of ion exchange materials. Journal of chemistry 2013

[CR86] Kumar A, Thakur A, Panesar PS (2019) Lactic acid and its separation and purification techniques: A review. Reviews in Environmental Science Bio/Technology:1–31

[CR87] Kurzrock T, Weuster-Botz DJBl, (2010). Recovery of succinic acid from fermentation broth. Biotechnol Lett..

[CR88] Kwak H, Hwang DW, Hwang YK, Chang JS (2012). Recovery of alkyl lactate from ammonium lactate by an advanced precipitation process. Sep Purif Technol.

[CR89] Lateef H, Gooding A, Grimes S (2012). Use of 1-hexyl-3-methylimidazolium bromide ionic liquid in the recovery of lactic acid from wine. J Chem Technol Biotechnol.

[CR90] Lech M, Trusek A (2018). Batch electrodialysis of lactic acid obtained from LAB fermentation. Pol J Chem Technol.

[CR91] Li QZ, Jiang XL, Feng XJ, Wang JM, Sun C, Zhang HB, Xian M, Liu HZ (2016). Recovery processes of organic acids from fermentation broths in the biomass-based industry. J Microbiol Biotechnol.

[CR92] Litchfield JH, Neidleman SL, Laskin AI (1996). Microbiological production of lactic acid. Advances in applied microbiology.

[CR93] López-Garzón CS, Straathof AJJ (2014). Recovery of carboxylic acids produced by fermentation. Biotechnol Adv.

[CR94] Lu Z, Lu M, He F, Yu L (2009). An economical approach for d-lactic acid production utilizing unpolished rice from aging paddy as major nutrient source. Biores Technol.

[CR95] Luca C (2000) Organic Ion Exchangers. Encyclopedia of Separation Science:1617

[CR96] Lunelli BH, Andrade RR, Atala DI, Maciel MRW, Maugeri Filho F, Maciel Filho R (2010). Production of lactic acid from sucrose: strain selection, fermentation, and kinetic modeling. Appl Biochem Biotechnol.

[CR97] Luongo V, Palma A, Rene ER, Fontana A, Pirozzi F, Esposito G, Lens PN (2019). Lactic acid recovery from a model of* Thermotoga neapolitana* fermentation broth using ion exchange resins in batch and fixed-bed reactors. Sep Sci Technol.

[CR98] Malav MK, Prasad S, Kharia SK, Kumar S, Sheetal K, Kannojiya S (2017). Furfural and 5-HMF: Potent fermentation inhibitors and their removal techniques. Int J Curr Microbiol Appl Sci.

[CR99] Mandegari MA, Farzad S, van Rensburg E, Görgens JF (2017). Multi-criteria analysis of a biorefinery for co-production of lactic acid and ethanol from sugarcane lignocellulose. Biofuels, Bioproducts Biorefining.

[CR100] Mao F, Zhang G, Tong J, Xu T, Wu Y (2014). Anion exchange membranes used in diffusion dialysis for acid recovery from erosive and organic solutions. Sep Purif Technol.

[CR101] Marceau A, Zagorec M, Champomier-Verges M (2002) Analysis of *Lactobacillus sakei* adaptation to its environment by a proteomic approach. Sciences des Aliments (France).

[CR102] Marinova N, Yankov D (2009) Toxicity of some solvents and extractants towards *Lactobacillus casei* cells. Bulg Chem Commun[online] 41:368–373

[CR103] Martinez FAC, Balciunas EM, Salgado JM, González JMD, Converti A, de Souza Oliveira RP (2013). Lactic acid properties, applications and production: A review. Trends Food Sci Technol.

[CR104] Matsumoto M, Nishimura M, Kobayashi H, Kondo K (2016). Extractive fermentation of lactic acid with Hiochi bacteria in a two-liquid phase system. Ferment Technol.

[CR105] Moldes A, Alonso J, Parajo J (2003). Recovery of lactic acid from simultaneous saccharification and fermentation media using anion exchange resins. Bioprocess Biosyst Eng.

[CR106] Monteagudo JM, Aldavero M (1999). Production of l-lactic acid by Lactobacillus delbrueckii in chemostat culture using an ion exchange resins system. J Chem Technol Biotechnol Int Res Process, Environ Clean Technol.

[CR107] Nair NR, Nampoothiri KM, Banarjee R, Reddy G (2016). Simultaneous saccharification and fermentation (SSF) of jackfruit seed powder (JFSP) to  l-lactic acid and to polylactide polymer. Biores Technol.

[CR108] Nam HG, Park KM, Lim SS, Mun S (2011). Adsorption equilibria of succinic acid and lactic acid on Amberchrom CG300C resin. J Chem Eng Data.

[CR109] Nancib A, Nancib N, Boubendir A, Boudrant J (2015). The use of date waste for lactic acid production by a fed-batch culture using* Lactobacillus casei* subsp. rhamnosus. Brazilian J Microbiol.

[CR110] Neu AK, Pleissner D, Mehlmann K, Schneider R, Puerta-Quintero GI, Venus J (2016). Fermentative utilization of coffee mucilage using Bacillus coagulans and investigation of down-stream processing of fermentation broth for optically pure l (+)-lactic acid production. Biores Technol.

[CR111] Nielsen DR, Amarasiriwardena GS, Prather KL (2010). Predicting the adsorption of second generation biofuels by polymeric resins with applications for in situ product recovery (ISPR). Biores Technol.

[CR112] Okeola F, Odebunmi E (2010). Freundlich and Langmuir isotherms parameters for adsorption of methylene blue by activated carbon derived from agrowastes. Adv Natural Appl Sci.

[CR113] Oliveira FS, Araújo JM, Ferreira R, Rebelo LPN, Marrucho IM (2012). Extraction of  l-lactic, l-malic, and succinic acids using phosphonium-based ionic liquids. Sep Purif Technol.

[CR114] Olszewska-Widdrat A, Alexandri M, López-Gómez JP, Schneider R, Mandl M, Venus J (2019). Production and purification of l-lactic acid in lab and pilot scales using sweet sorghum juice. Fermentation.

[CR115] Oonkhanond B, Jonglertjunya W, Srimarut N, Bunpachart P, Tantinukul S, Nasongkla N, Sakdaronnarong C (2017). Lactic acid production from sugarcane bagasse by an integrated system of lignocellulose fractionation, saccharification, fermentation, and ex-situ nanofiltration. J Environ Chem Eng.

[CR116] Othman M, Ariff AB, Rios-Solis L, Halim M (2017). Extractive fermentation of lactic acid in lactic acid bacteria cultivation: A review. Front Microbiol.

[CR117] Othman M, Ariff AB, Wasoh H, Kapri MR, Halim M (2017). Strategies for improving production performance of probiotic Pediococcus acidilactici viable cell by overcoming lactic acid inhibition. AMB Express.

[CR118] Othman M, Ariff AB, Kapri MR, Rios Solis L, Halim M (2018). Growth enhancement of probiotic* Pediococcus acidilactici* by extractive fermentation of lactic acid exploiting anion-exchange resin. Front Microbiol.

[CR119] Pal P, Dey P Developing a sustainable technology for clean production of lactic acid. In: International Conference on Chemical, Ecology and Environmental Sciences (ICEES’2012) March, Bangkok, 2012. vol 17. p e18

[CR120] Pal P, Sikder J, Roy S, Giorno L (2009). Process intensification in lactic acid production: A review of membrane based processes. Chem Eng Process.

[CR121] Park C, Nam HG, Lee KB, Mun S (2014). Optimal design and experimental validation of a simulated moving bed chromatography for continuous recovery of formic acid in a model mixture of three organic acids from Actinobacillus bacteria fermentation. J Chromatogr A.

[CR122] Patel M, Bassi AS, Zhu JJX, Gomaa H (2008). Investigation of a dual-particle liquid–solid circulating fluidized bed bioreactor for extractive fermentation of lactic acid. Biotechnol Prog.

[CR123] Plavec TV, Berlec A (2020). Safety aspects of genetically modified lactic acid bacteria. Microorganisms.

[CR124] Pleissner D, Schneider R, Venus J, Koch T (2017). Separation of lactic acid and recovery of salt-ions from fermentation broth. J Chem Technol Biotechnol.

[CR125] Pradhan N, Rene E, Lens P, Dipasquale L, D’Ippolito G, Fontana A, Panico A, Esposito G (2017). Adsorption behaviour of lactic acid on granular activated carbon and anionic resins: thermodynamics, isotherms and kinetic studies. Energies.

[CR126] Prückler M, Lorenz C, Endo A, Kraler M, Dürrschmid K, Hendriks K, da Silva FS, Auterith E, Kneifel W, Michlmayr H (2015). Comparison of homo- and heterofermentative lactic acid bacteria for implementation of fermented wheat bran in bread. Food Microbiol.

[CR127] Quintero J, Acosta A, Mejía C, Ríos R, Torres AM (2012). Purification of lactic acid obtained from a fermentative process of cassava syrup using ion exchange resins. Revista Facultad de Ingeniería Universidad de Antioquia.

[CR128] Ramos JL, García-Lorente F, Valdivia M, Duque E (2017). Green biofuels and bioproducts: bases for sustainability analysis. Microb Biotechnol.

[CR129] Rampai T, Thitiprasert S, Boonkong W, Kodama K, Tolieng V, Thongchul N (2016). Improved lactic acid productivity by simultaneous recovery during fermentation using resin exchanger. Asia-Pacific J Sci Technol.

[CR130] Rao VB, Kumar PS, Sailu C, Rao SRM (2014). Recovery of lactic acid by reactive distillation. J Appl Sci.

[CR131] Rincon J, Fuertes J, Rodriguez JF, Rodriguez L, Monteagudo JM (1997). Selection of a cation exchange resin to produce lactic acid solutions from whey fermentation broths. Solvent Extr Ion Exch.

[CR132] Rohm and Haas (2008). Ion Exchange for Dummies.

[CR133] Ruthven DM (1984). Principles of adsorption and adsorption processes.

[CR134] Bayazit SaS, İnci Is, Uslu H (2011). Adsorption of lactic acid from model fermentation broth onto activated carbon and amberlite IRA-67. J Chem Eng Data.

[CR135] Bayazit SaS, Uslu H, İnci Is (2011). Comparison of the efficiencies of amine extractants on lactic acid with different organic solvents. J Chem Eng Data.

[CR136] Schopmeyer HH, Arnold CR (1944) Lactic acid purification. US Patent 476,060, 6 June 1944

[CR137] Seeber G, Buchmeiser MR, Bonn GK, Bertsch T (1998). Determination of airborne, volatile amines from polyurethane foams by sorption onto a high-capacity cation-exchange resin based on poly (succinic acid). J Chromatogr A.

[CR138] Seong HA, Lee JS, Yoon SY, Song WY, Shin SJ (2016). Fermentation characteristics of acid hydrolysates by different neutralizing agents. Int J Hydrogen Energy.

[CR139] Serrazanetti DI, Gottardi D, Montanari C, Gianotti A (2013) Dynamic stresses of lactic acid bacteria associated to fermentation processes. In: Kongo JM (ed) Lactic Acid Bacteria-R & D for Food, Health and Livestock Purposes. IntechOpen, United Kingdom. 10.5772/51049

[CR140] Srivastava A, Roychoudhury PK, Sahai V (1992). Extractive lactic acid fermentation using ion-exchange resin. Biotechnol Bioeng.

[CR141] Syed Amran SN, Zainal Abidin N, Hashim H, Zubairi SI (2018). Saponin bitterness reduction of carica papaya leaf extracts through adsorption of weakly basic ion exchange resins. J Food Qual.

[CR142] Tabassum S (2019). A combined treatment method of novel mass bio system and ion exchange for the removal of ammonia nitrogen from micro-polluted water bodies. Chem Eng J.

[CR170] Timmer J, Van der Horst H, Robbertsen T (1993). Transport of lactic acid through reverse osmosis and nanofiltration membranes. J Membr Sci.

[CR143] Tong WY, Fu XY, Lee SM, Yu J, Liu JW, Wei DZ, Koo YM (2004). Purification of l (+)-lactic acid from fermentation broth with paper sludge as a cellulosic feedstock using weak anion exchanger Amberlite IRA-92. Biochem Eng J.

[CR144] Trikas ED, Papi RM, Kyriakidis DA, Zachariadis GA (2017). Evaluation of ion exchange and sorbing materials for their adsorption/desorption performance towards anthocyanins, total phenolics, and sugars from a grape pomace extract. Separations.

[CR145] Uslu H, Majumder S (2017). Adsorption studies of lactic acid by polymeric adsorbent amberlite XAD-7: equilibrium and kinetics. J Chem Eng Data.

[CR146] Uslu H, Datta D, Santos D, Bamufleh HS, Bayat C (2016). Separation of 2, 4, 6-trinitrophenol from aqueous solution by liquid–liquid extraction method: equilibrium, kinetics, thermodynamics and molecular dynamic simulation. Chem Eng J.

[CR147] Vandenberghe LP, Karp SG, de Oliveira PZ, de Carvalho JC, Rodrigues C, Soccol CR, Larroche C, Angeles M (2018). Solid-State Fermentation for the Production of Organic Acids. Current Developments in Biotechnology and Bioengineering.

[CR148] Vane LM (2005). A review of pervaporation for product recovery from biomass fermentation processes. J Chem Technol Biotechnol Int Res Process Environ Clean Technol.

[CR149] Wang X, Wang Y, Zhang X, Feng H, Xu T (2013). In-situ combination of fermentation and electrodialysis with bipolar membranes for the production of lactic acid: continuous operation. Biores Technol.

[CR150] Wang C, Li Q, Wang D, Xing J (2014). Improving the lactic acid production of Actinobacillus succinogenes by using a novel fermentation and separation integration system. Process Biochem.

[CR151] Wasewar KL (2005). Separation of lactic acid: Recent advances. Chem Biochem Eng Q.

[CR152] Wasewar KL, Pangarkar VG, Heesink ABM, Versteeg GF (2003). Intensification of enzymatic conversion of glucose to lactic acid by reactive extraction. Chem Eng Sci.

[CR153] Wasewar KL, Yawalkar AA, Moulijn JA, Pangarkar VG (2004). Fermentation of glucose to lactic acid coupled with reactive extraction: a review. Ind Eng Chem Res.

[CR154] Wee YJ, Kim JN, Ryu HW (2006). Biotechnological production of lactic acid and its recent applications. Food Technol Biotechnol.

[CR155] Wojtyniak B, Kołodziejczyk J, Szaniawska D (2016). Production of lactic acid by ultrafiltration of fermented whey obtained in bioreactor equipped with ZOSS membrane. Chem Eng J.

[CR156] Xu S, Lan K, Li J, He T, Hu C (2018). Separation of lactic acid from synthetic solutions and the mixture directly derived from corn stover by aqueous two phase extraction. Sep Purif Technol.

[CR157] Yang W, Li A, Ce Fu, Fan J, Zhang Q (2007). Adsorption mechanism of aromatic sulfonates onto resins with different matrices. Ind Eng Chem Res.

[CR158] Yankov D, Molinier J, Kyuchoukov G, Albet J, Malmary G (2005). Improvement of the lactic acid extraction. Extraction from aqueous solutions and simulated fermentation broth by means of mixed extractant and TOA, partially loaded with HCl. Chem Biochem Eng Q.

[CR159] Yee YY, Ching YC, Rozali S, Hashim NA, Singh R (2016). Preparation and characterization of poly (lactic acid)-based composite reinforced with oil palm empty fruit bunch fiber and nanosilica. BioResources.

[CR160] Yousuf A, Bonk F, Bastidas-Oyanedel JR, Schmidt JE (2016). Recovery of carboxylic acids produced during dark fermentation of food waste by adsorption on Amberlite IRA-67 and activated carbon. Biores Technol.

[CR161] Yuwono SD, Ghofar A, Kokugan T (2008). Effect of product inhibitions on L-lactic acid fermentation from fresh cassava roots in tofu liquid waste by *Streptococcus bovis*. Japan Journal of Food Engineering.

[CR162] Yuwono SD, Nugroho RH, Mulyono B, Suharso SI (2017). Purification of lactic acid from cassava bagasse fermentation using ion exchange. ARPN J Engin Appl Sci.

[CR163] Zaini NABM, Chatzifragkou A, Charalampopoulos D (2019). Microbial production of d-lactic acid from dried distiller's grains with solubles. Eng Life Sci.

[CR164] Zaini NAM, Chatzifragkou A, Tverezovskiy V, Charalampopoulos D (2019b) Purification and polymerisation of microbial* d*-lactic acid from DDGS hydrolysates fermentation. Biochemical Engineering Journal:107265

[CR165] Zhang K, Yang ST (2015). In situ recovery of fumaric acid by intermittent adsorption with IRA-900 ion exchange resin for enhanced fumaric acid production by Rhizopus oryzae. Biochem Eng J.

[CR166] Zhang Y, Vadlani PV, Kumar A, Hardwidge PR, Govind R, Tanaka T, Kondo A (2016). Enhanced d-lactic acid production from renewable resources using engineered* Lactobacillus plantarum*. Appl Microbiol Biotechnol.

[CR167] Zhang Y, Qian Z, Liu P, Liu L, Zheng Z, Ouyang J (2018). Efficient in situ separation and production of l-lactic acid by Bacillus coagulans using weak basic anion-exchange resin. Bioprocess Biosyst Eng.

[CR168] Zhang YP, Adi VSK, Huang HL, Lin HP, Huang ZH (2019). Adsorption of metal ions with biochars derived from biomass wastes in a fixed column: adsorption isotherm and process simulation. J Ind Eng Chem.

[CR169] Zhang Y, Hu Y, Wang L, Sun W (2019). Systematic review of lithium extraction from salt-lake brines via precipitation approaches. Miner Eng.

